# Publisher Correction: Patterns in soil microbial diversity across Europe

**DOI:** 10.1038/s41467-023-39596-x

**Published:** 2023-07-18

**Authors:** Maëva Labouyrie, Cristiano Ballabio, Ferran Romero, Panos Panagos, Arwyn Jones, Marc W. Schmid, Vladimir Mikryukov, Olesya Dulya, Leho Tedersoo, Mohammad Bahram, Emanuele Lugato, Marcel G. A. van der Heijden, Alberto Orgiazzi

**Affiliations:** 1 Department of Plant and Microbial Biology, University of Zurich, Zürich, Switzerland; 2 European Commission, Joint Research Centre (JRC), Ispra, VA Italy; 3 Plant-Soil-Interactions, Research Division Agroecology and Environment, Agroscope, Zürich, Switzerland; 4 MWSchmid GmbH, Glarus, Switzerland; 5 Mycology and Microbiology Center, University of Tartu, Tartu, Estonia; 6 Department of Botany, Institute of Ecology and Earth Sciences, University of Tartu, Tartu, Estonia; 7 Department of Ecology, Swedish University of Agricultural Sciences, Uppsala, Sweden

**Keywords:** Biodiversity, Microbial ecology, Biodiversity, Microbial ecology

Correction to: *Nature Communications* 10.1038/s41467-023-37937-4, published online 08 June 2023

The original version of this Article contained an error in Fig. 1a, in which part of the ‘LUCAS Vegetation cover’ legend was omitted. This has now been corrected in the PDF and HTML versions of the Article.

