# Supplementary material for: Patterns in soil microbial diversity across Europe
Source: Nat Commun. 2023 Jun 8;14:3311. doi: 10.1038/s41467-023-37937-4 (PMC10250377; doi:10.1038/s41467-023-37937-4)
Supplement: Supplementary file 1 — Supplementary Information file [file 41467_2023_37937_MOESM1_ESM.pdf]

Supplementary Materials for

Patterns in soil microbial diversity across Europe

Maëva Labouyrie, Cristiano Ballabio, Ferran Romero, Panos Panagos, Arwyn Jones, Marc W. Schmid, Vladimir Mikryukov, Olesya Dulya, Leho Tedersoo, Mohammad Bahram, Emanuele Lugato, Marcel G.A. van der Heijden*, Alberto Orgiazzi*

*Corresponding authors:alberto.orgiazzi@gmail.com and marcel.vanderheijden@agroscope.admin.ch

This PDF file includes:

Supplementary Fig. 1 to 17
Supplementary Tables 1 to 7

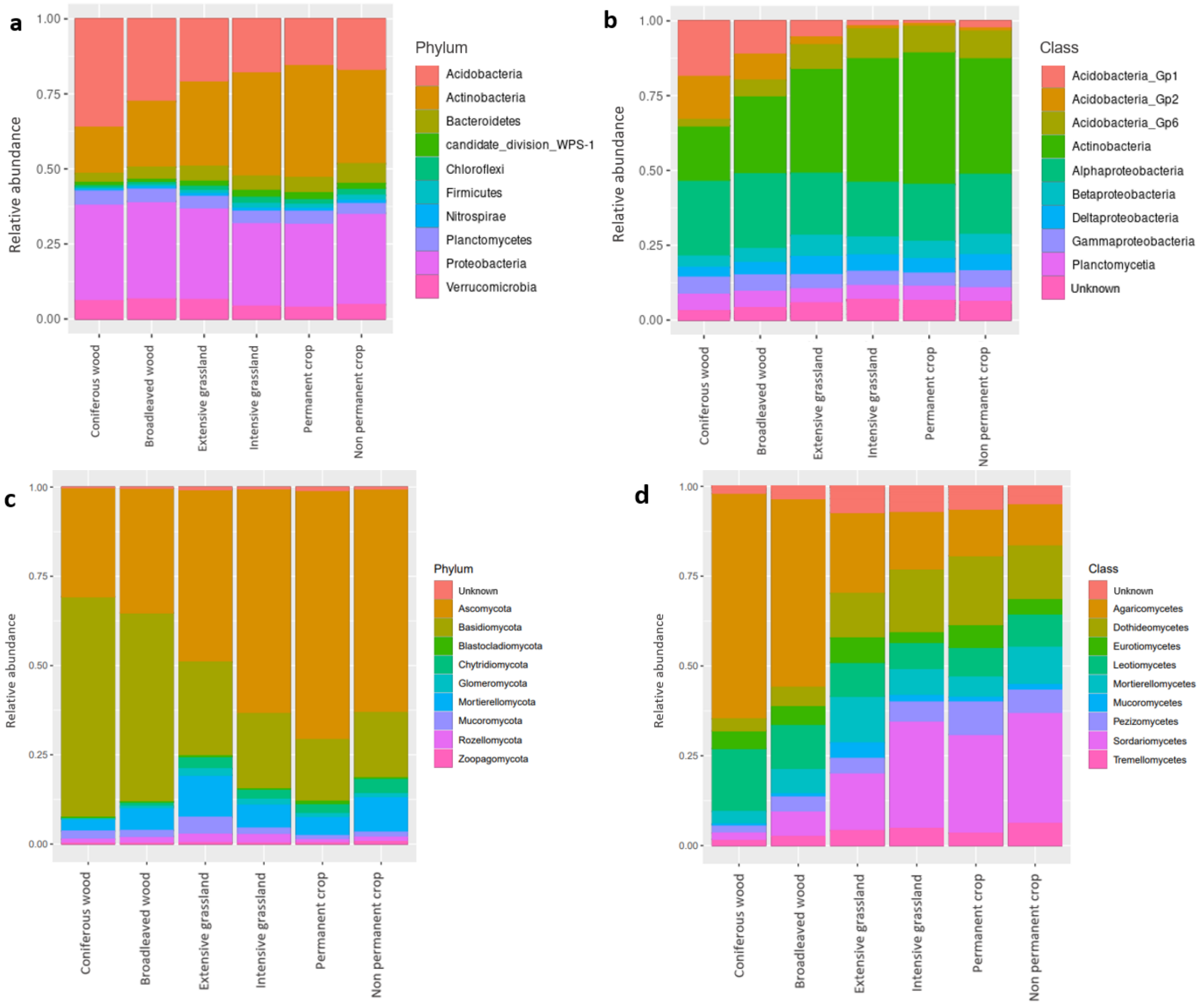

Supplementary Fig. 1.

Mean relative abundances of the ten most abundant **a** bacterial phyla, **b** fungal phyla, **c** bacterial classes, **d** fungal classes at the communtity-level. Different colours correspond to different bacterial/fungal phyla/classes. Source data are provided as a Source Data file.

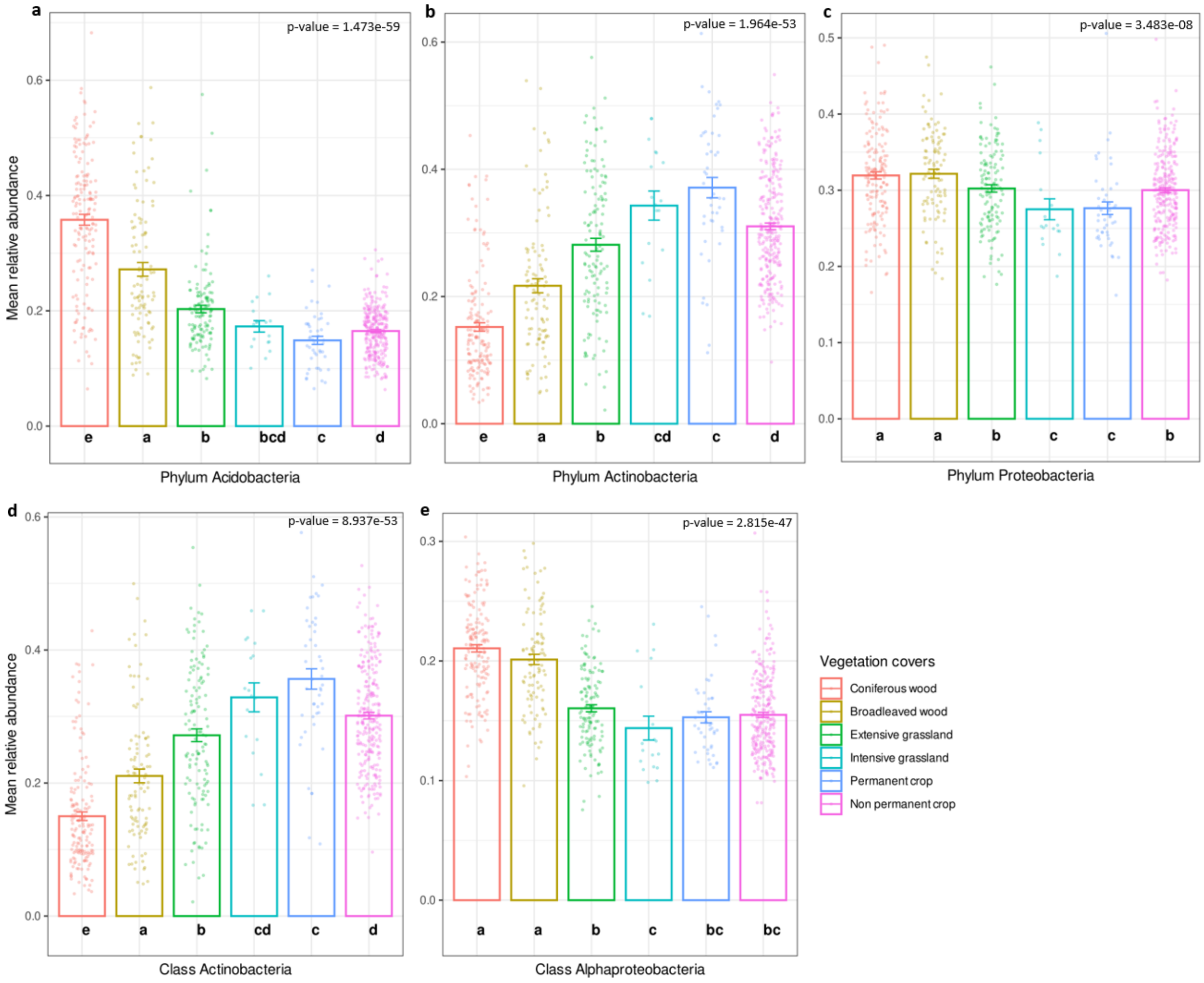

Supplementary Fig. 2.

Mean relative abundances (± SE) of bacterial phyla and classes which mean relative abundance > 10% at the community-level. **a** Phylum Acidobacteria. **b** Phylum Actinobacteria. **c** Phylum Proteobacteria. **c** Class Actinobacteria. **d** Class Alphaproteobacteria. Error bars represent standard error. Different letters correspond to a significant difference among relative abundance of a phylum (or class) in compared vegetation cover types, and p-value corresponds to the one obtained with a Kruskal-Wallis test testing the vegetation cover effect. Here, colours represent the different vegetation cover types and n = 715 total sites, with 160 belonging to coniferous woods, 99 to broadleaved woods, 128 to extensive grasslands, 18 to intensive grasslands, 46 to permanent crops and 264 to non-permanent crops sites. Source data are provided as a Source Data file.

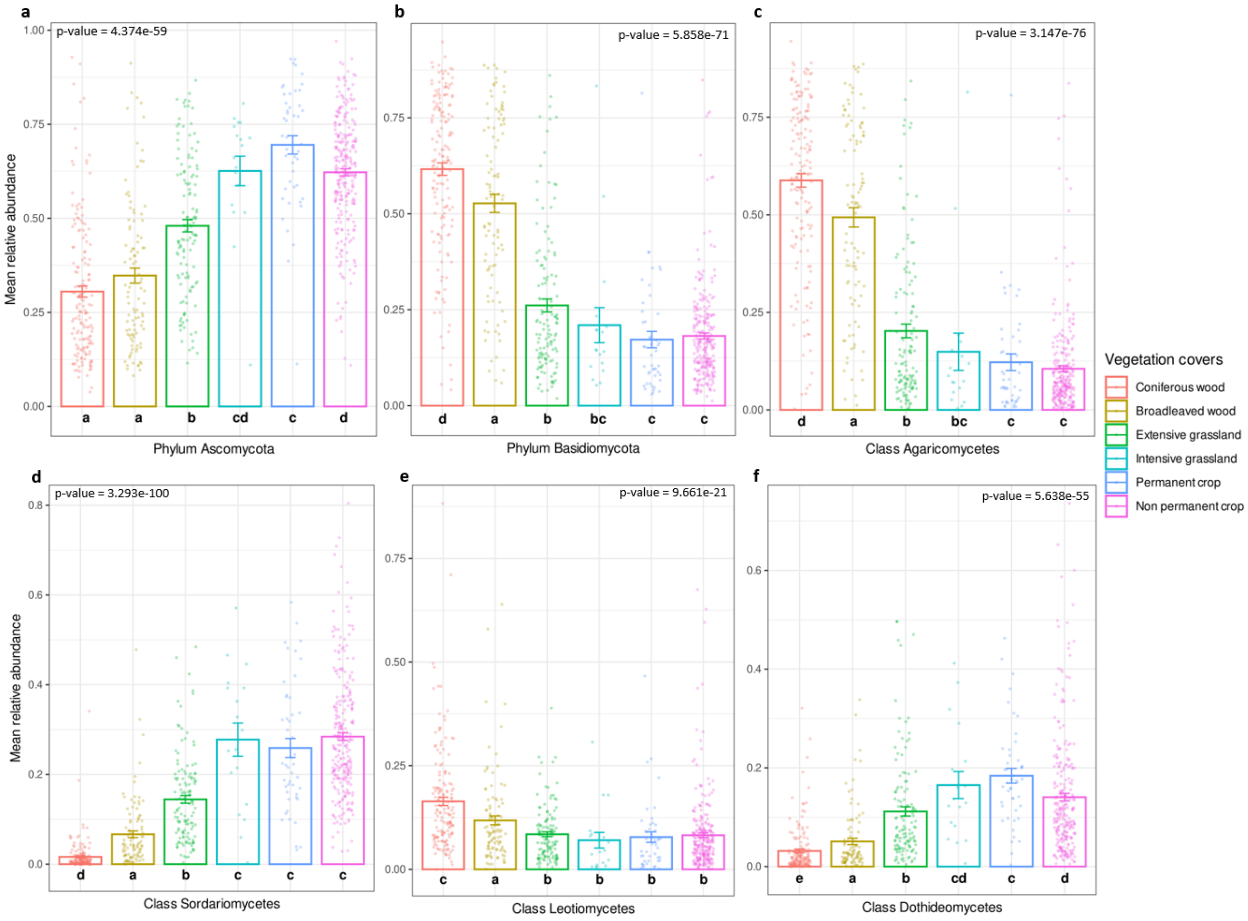

Supplementary Fig. 3.

Mean relative abundances (± SE) of fungal phyla and classes which mean relative abundance > 10% at the community-level. **a** Phylum Ascomycota. **b** Phylum Basidiomycota. **c** Class Agaricomycetes. **d** Class Sordariomycetes. **e** Class Leotiomycetes. **f** Class Dothideomycetes. Error bars represent standard error. Different letters correspond to a significant difference among relative abundance of a phylum (or class) in compared vegetation cover types, and p-value corresponds to the one obtained with a Kruskal-Wallis test testing the vegetation cover effect. Here, colours represent the different vegetation cover types and n = 715 total sites, with 160 belonging to coniferous woods, 99 to broadleaved woods, 128 to extensive grasslands, 18 to intensive grasslands, 46 to permanent crops and 264 to non-permanent crops sites. Source data are provided as a Source Data file.

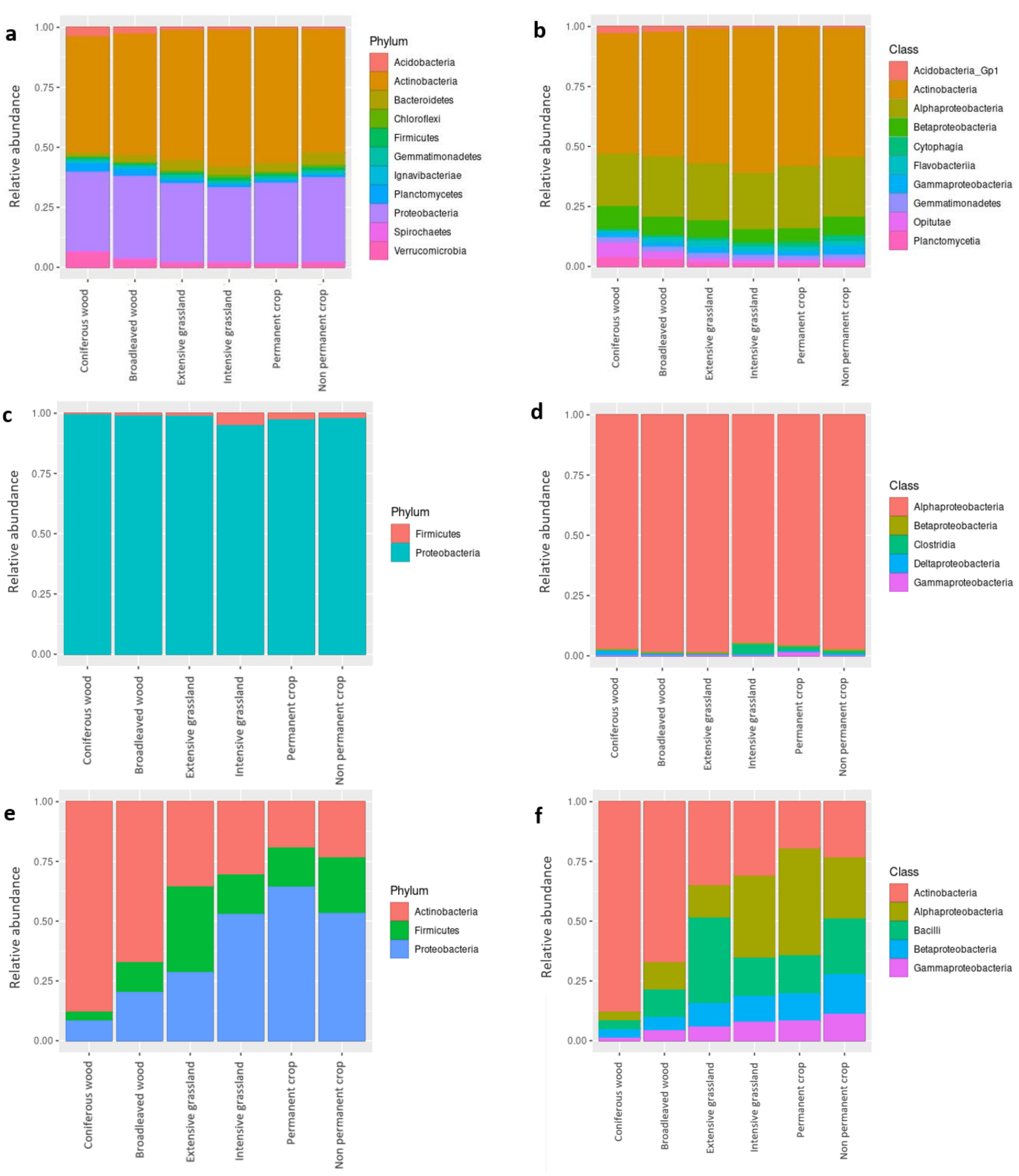

Supplementary Fig. 4.

Mean relative abundances of bacterial phyla and classes at the functional group-level for **a** bacterial chemoheterotrophs phyla, **b** bacterial chemoheterotrophs classes, **c** bacterial N-fixers phyla, **d** bacterial N-fixers classes, **e** bacterial pathogens phyla and **f** bacterial pathogens classes. Different colours correspond to different bacterial/fungal phyla/classes. Source data are provided as a Source Data file.

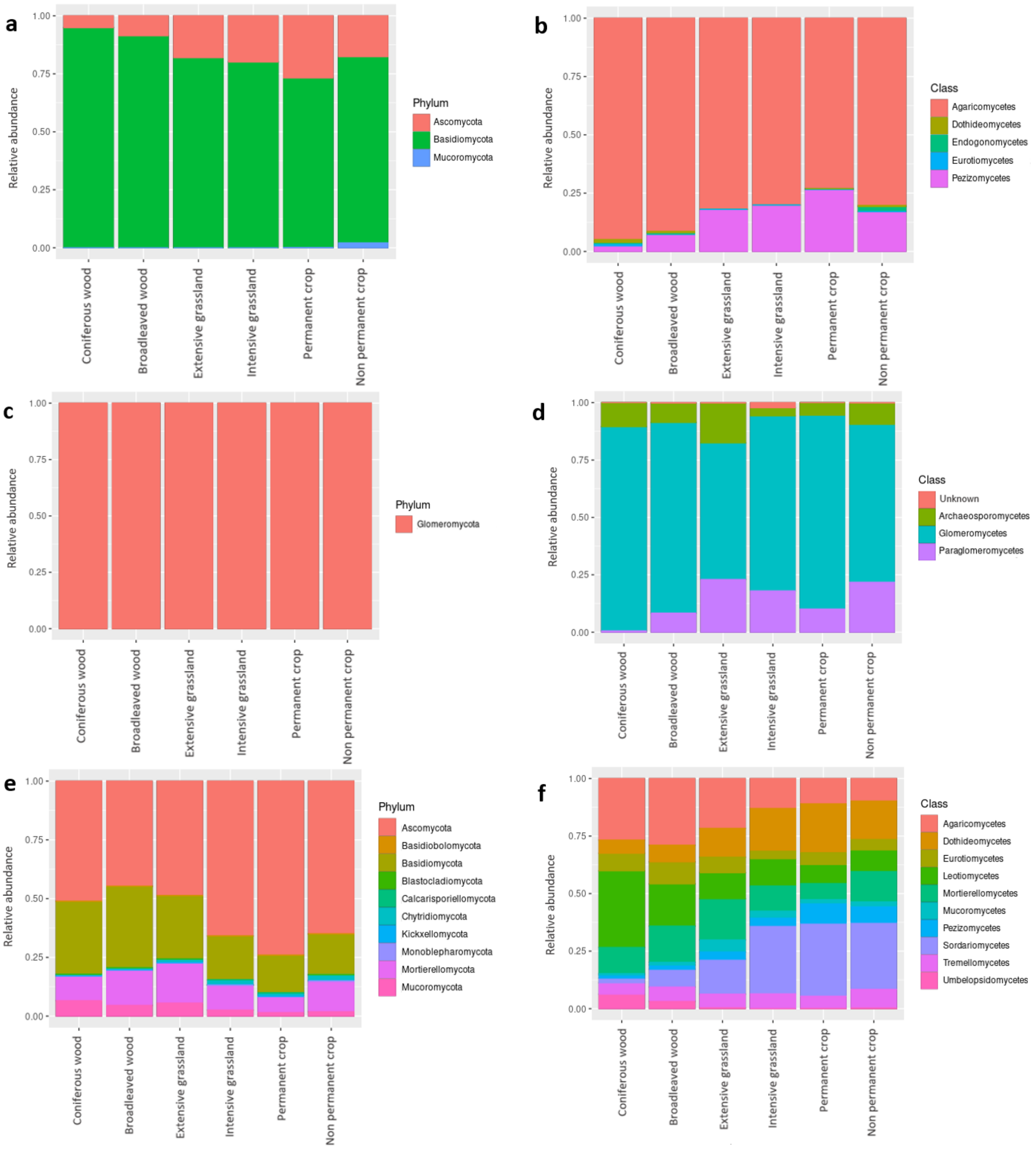

a
b
c
d
e
f
Relative abundance
1.00
0.75
0.50
0.25
0.00
Coniferous wood
Broadleaved wood
Extensive grassland
Intensive grassland
Permanent crop
Non permanent crop
Phylum
Ascomycota
Basidiomycota
Mucoromycota
Class
Agaricomycetes
Dothideomycetes
Endogonomycetes
Eurotiomycetes
Pezizomycetes
Phylum
Glomeromycota
Class
Unknown
Archaeosporomycetes
Glomeromycetes
Paraglomeromycetes
Phylum
Ascomycota
Basidiobolomycota
Basidiomycota
Blastocladiomycota
Calcarisporiellomycota
Chytridiomycota
Kickxellomycota
Monoblepharomycota
Mortierellomycota
Mucoromycota
Class
Agaricomycetes
Dothideomycetes
Eurotiomycetes
Leotiomycetes
Mortierellomycetes
Mucoromycetes
Pezizomycetes
Sordariomycetes
Tremellomycetes
Umbelopsidomycetes

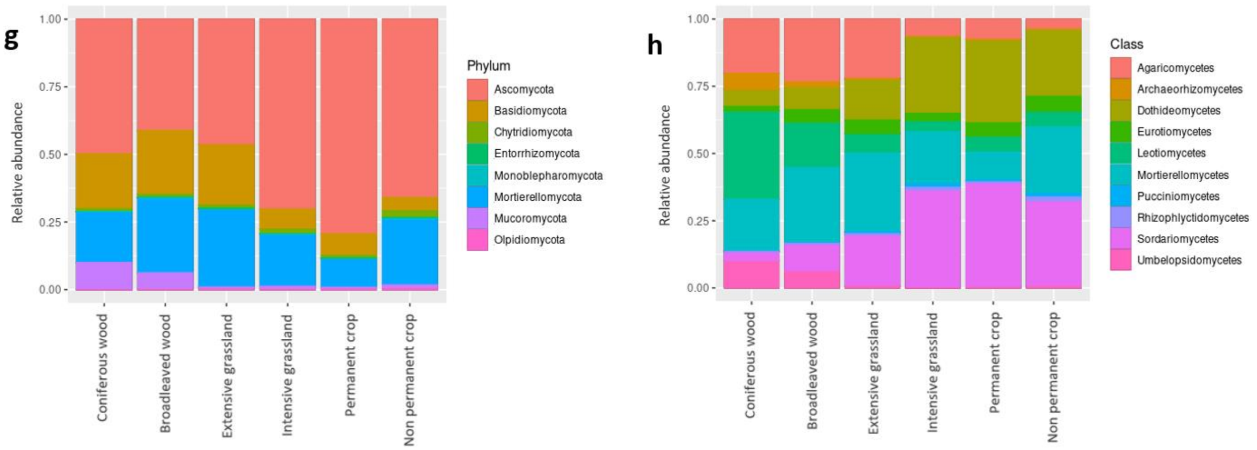

Supplementary Fig. 5. Mean relative abundances of fungal phyla and classes at the functional group-level for **a** ectomycorrhizal fungi phyla, **b** ectomycorrhizal fungi classes, **c** arbuscular mycorrhizal fungi phyla, **d** arbuscular mycorrhizal classes, **e** fungal saprotrophs ten most abundant phyla, **f** fungal saprotrophs ten most abundant classes, **g** fungal pathogens ten most abundant phyla and **h** fungal pathogens ten most abundant classes. Different colours correspond to different bacterial/fungal phyla/classes. Source data are provided as a Source Data file.

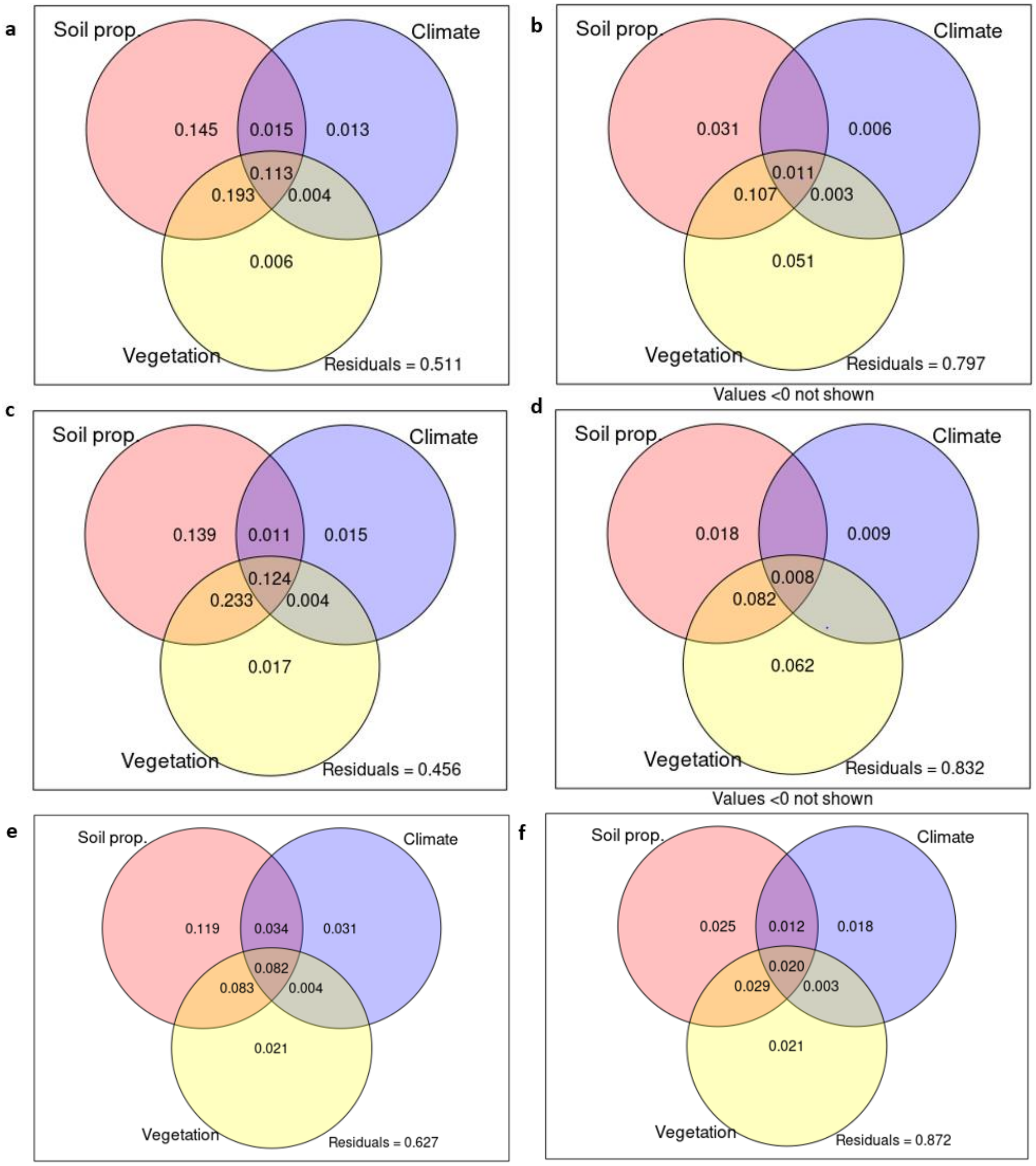

Supplementary Fig. 6.

Variation partitioning plots testing for the effect of soil properties, vegetation cover and climate on **a** bacterial observed richness, **b** fungal observed richness, **c** bacterial Shannon index, **d** fungal Shannon index, **e** bacterial beta-diversity and **f** fungal beta-diversity. Beta diversity corresponds to the Bray-Curtis dissimilarity matrix calculated on the Hellinger-transformed sample-by-(z)OTU table. Here soil properties and climate correspond to the variables preselected in the models. Source data are provided as a Source Data file.

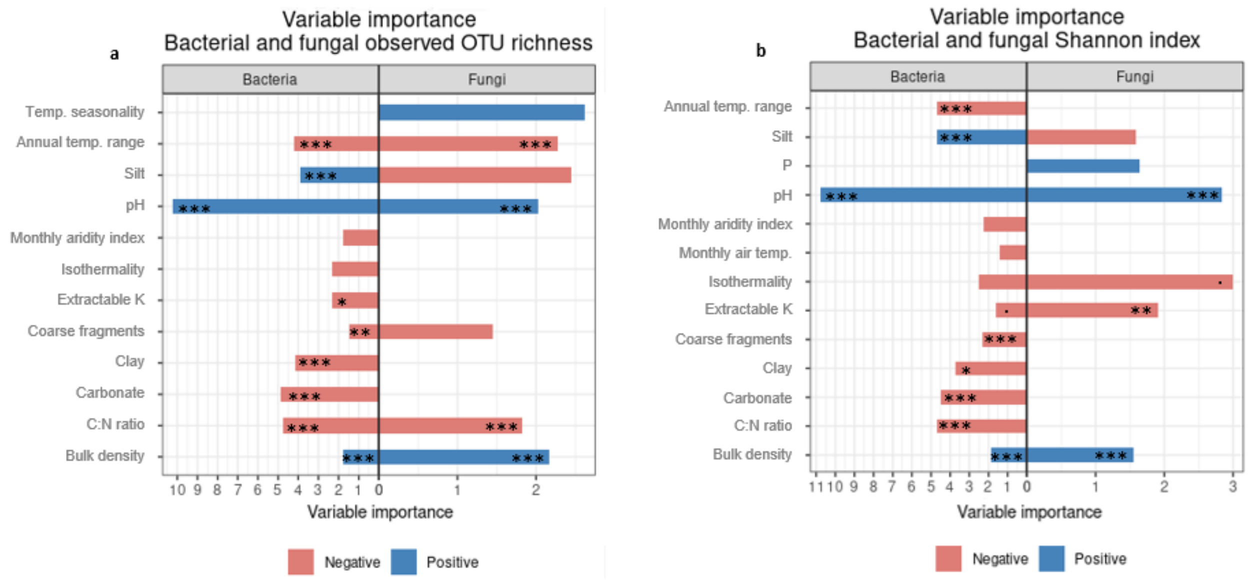

Supplementary Fig. 7.

a Variable importance for each numerical (soil or climatic) property in explaining bacterial and fungal observed richness. **b** Variable importance for each numerical (soil or climatic) property in explaining bacterial and fungal Shannon index (right). Only the numerical properties selected by the models are presented, but the vegetation cover was selected as a significant important (categorical) variable as well and included in the models. Colours represent the positive or negative sign of the variable in the linear model found after feature selection. The stars represent the level of significance of the p-value for each term in the one-way ANOVA (*** $p < 0.001$; ** $p < 0.01$; * $p < 0.05$; . $p < 0.1$). Exact p-values are provided in Supplementary Data file 2. Source data are provided as a Source Data file.

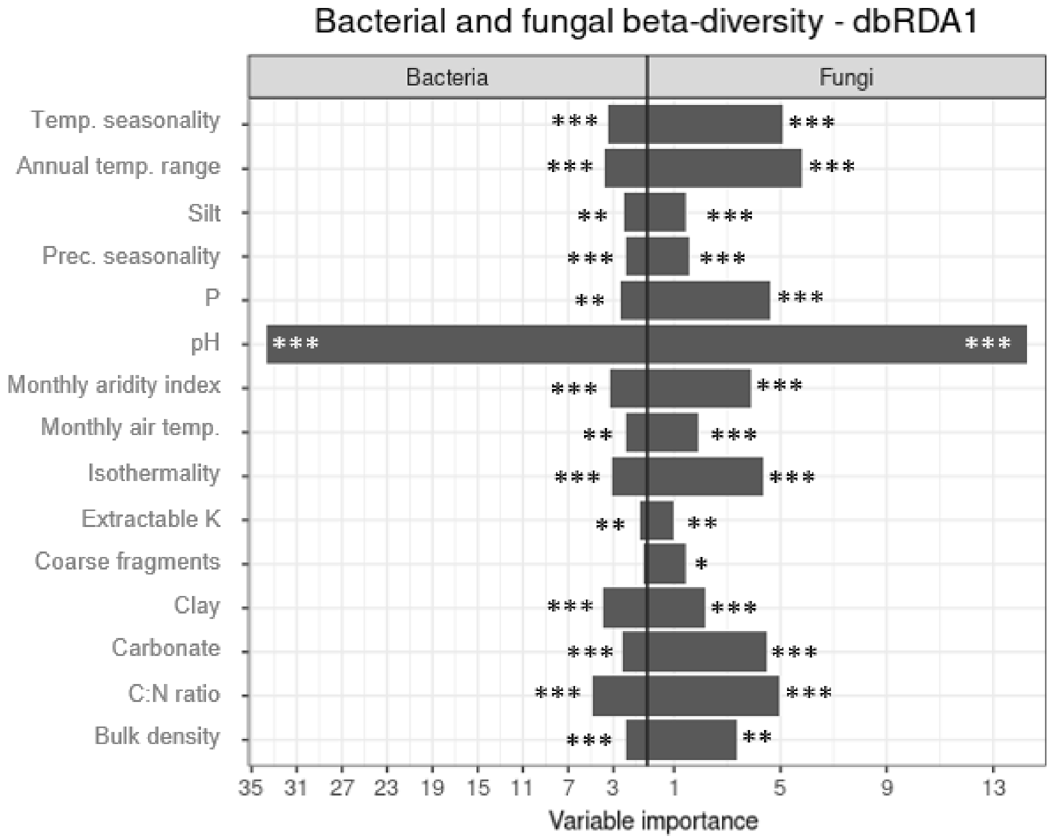

Supplementary Fig. 8.

Variable importance for each numerical (soil or climatic) property in explaining bacterial and fungal beta-diversity, calculated on the scores of the first axis (dbRDA1) of the bacterial or fungal dbRDA. Only the numerical properties selected by the models are presented, but the vegetation cover was selected as a significant important (categorical) variable as well and included in the models. The stars represent the level of significance of the p-value for each term in the one-way ANOVA (*** $p < 0.001$; ** $p < 0.01$; * $p < 0.05$; . $p < 0.1$). Exact p-values are provided in Supplementary Data file 2. Source data are provided as a Source Data file.

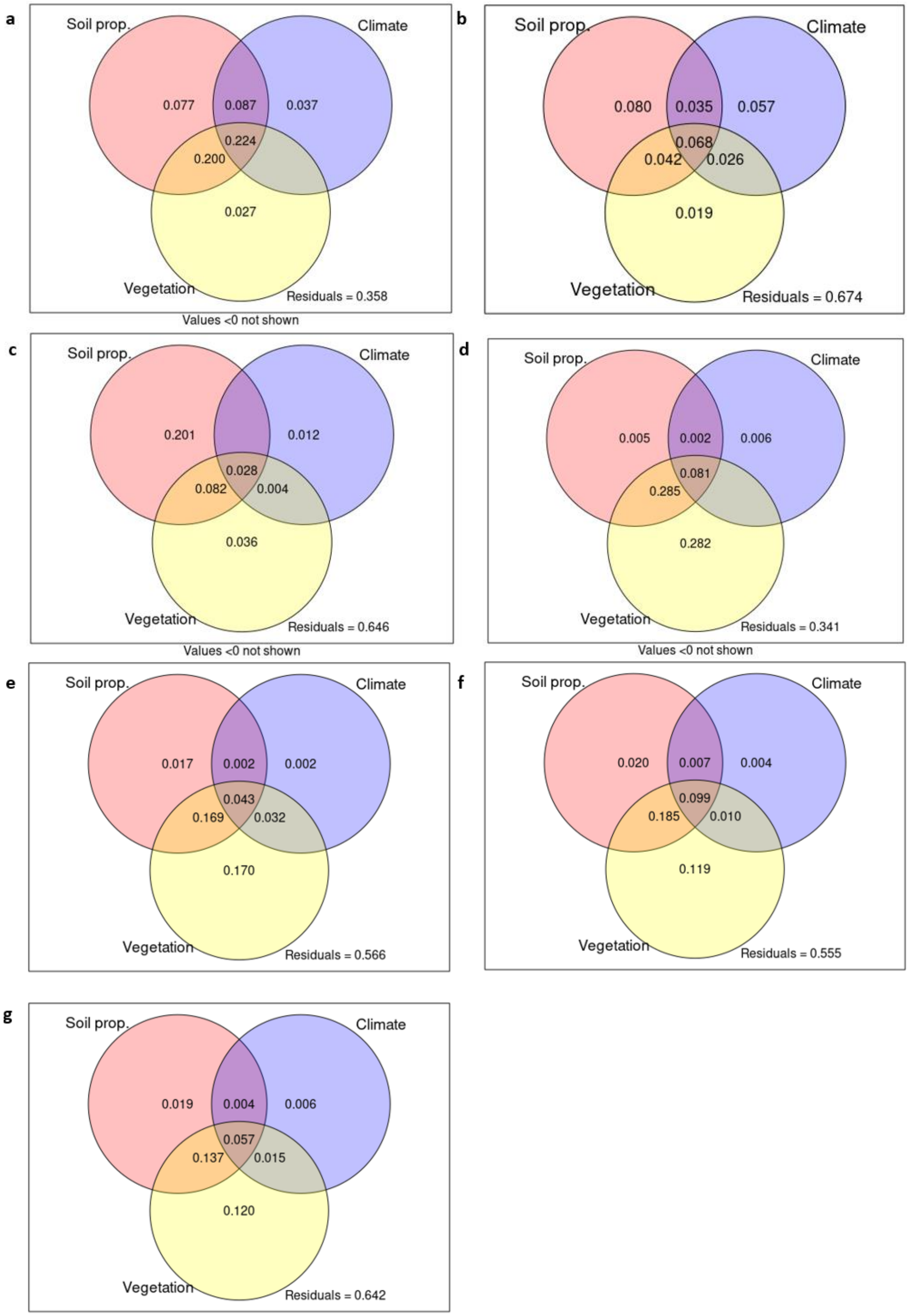

Supplementary Fig. 9.

Variation partitioning plots testing for the effect of soil properties, vegetation cover and climate on bacterial and fungal functional groups for **a** bacterial chemoheterotrophs, **b** bacterial N-fixers, **c** bacterial pathogens, **d** ectomycorrhizal fungi, **e** arbuscular mycorrhizal fungi, **f** fungal saprotrophs and **g** fungal plant pathogens. Here soil properties and climate correspond to the variables preselected in the models. Source data are provided as a Source Data file.

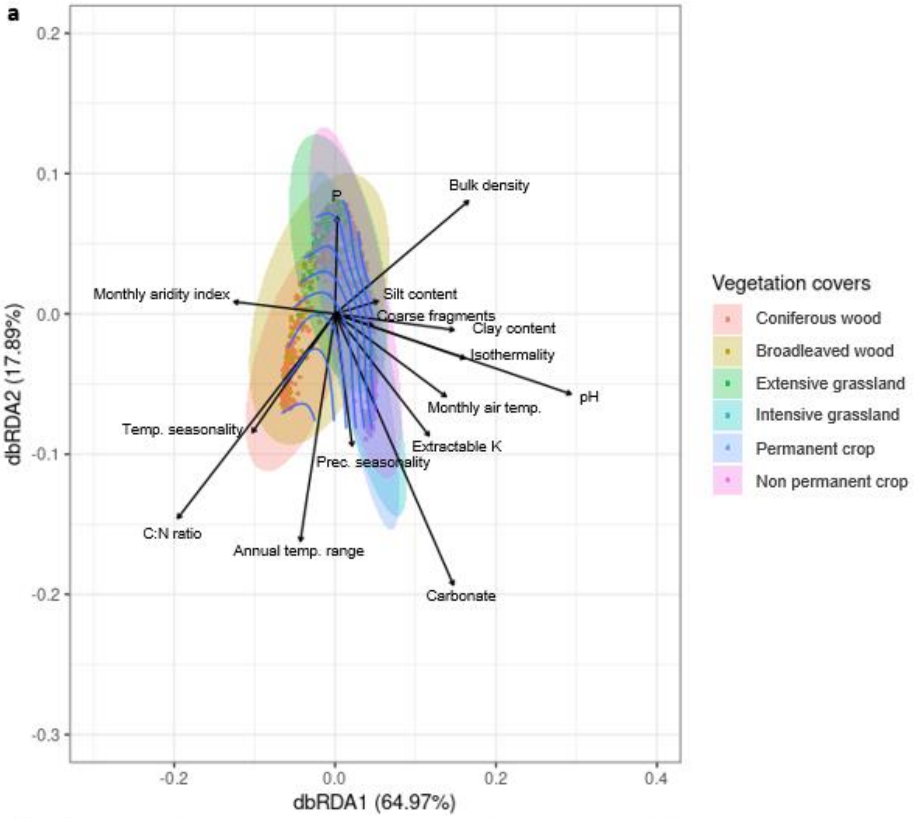
a
Bulk density
P
Monthly aridity index
Silt content
Coarse fragments
Clay content
Isothermality
pH
Monthly air temp.
Extractable K
Prec. seasonality
Temp. seasonality
C:N ratio
Annual temp. range
Carbonate
dbRDA2 (17.89%)
dbRDA1 (64.97%)
0.2
0.1
0.0
-0.1
-0.2
-0.3
-0.2
0.0
0.2
0.4
Vegetation covers
Coniferous wood
Broadleaved wood
Extensive grassland
Intensive grassland
Permanent crop
Non permanent crop

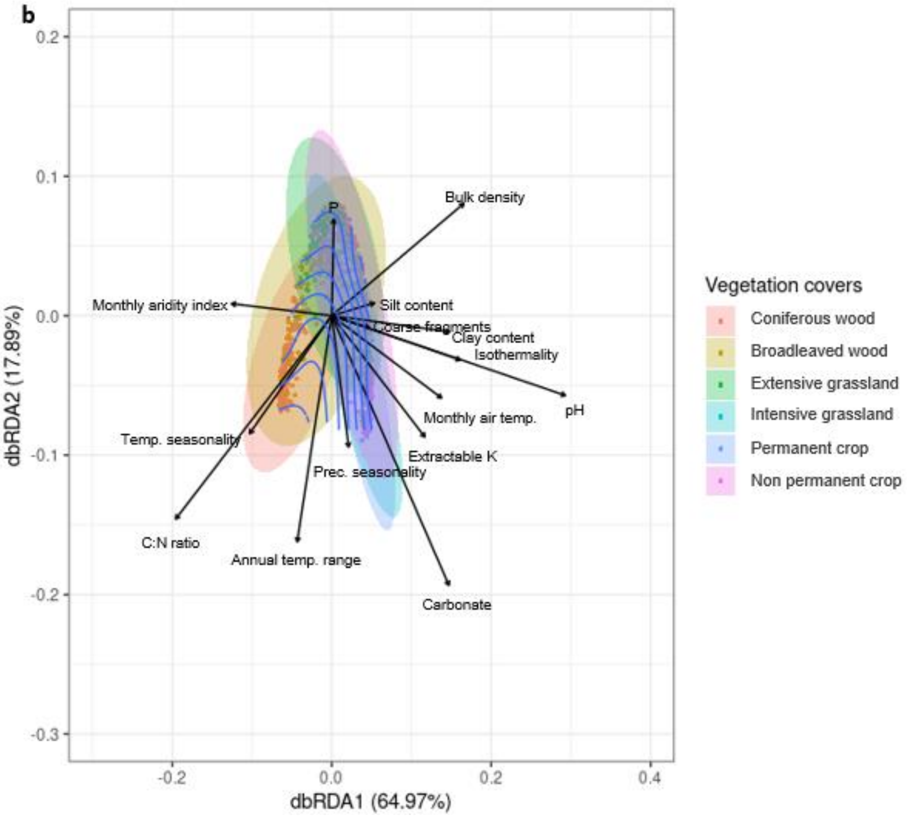
b
Bulk density
P
Monthly aridity index
Silt content
Coarse fragments
Clay content
Isothermality
pH
Monthly air temp.
Extractable K
Prec. seasonality
Temp. seasonality
C:N ratio
Annual temp. range
Carbonate
dbRDA2 (17.89%)
dbRDA1 (64.97%)
0.2
0.1
0.0
-0.1
-0.2
-0.3
-0.2
0.0
0.2
0.4
Vegetation covers
Coniferous wood
Broadleaved wood
Extensive grassland
Intensive grassland
Permanent crop
Non permanent crop

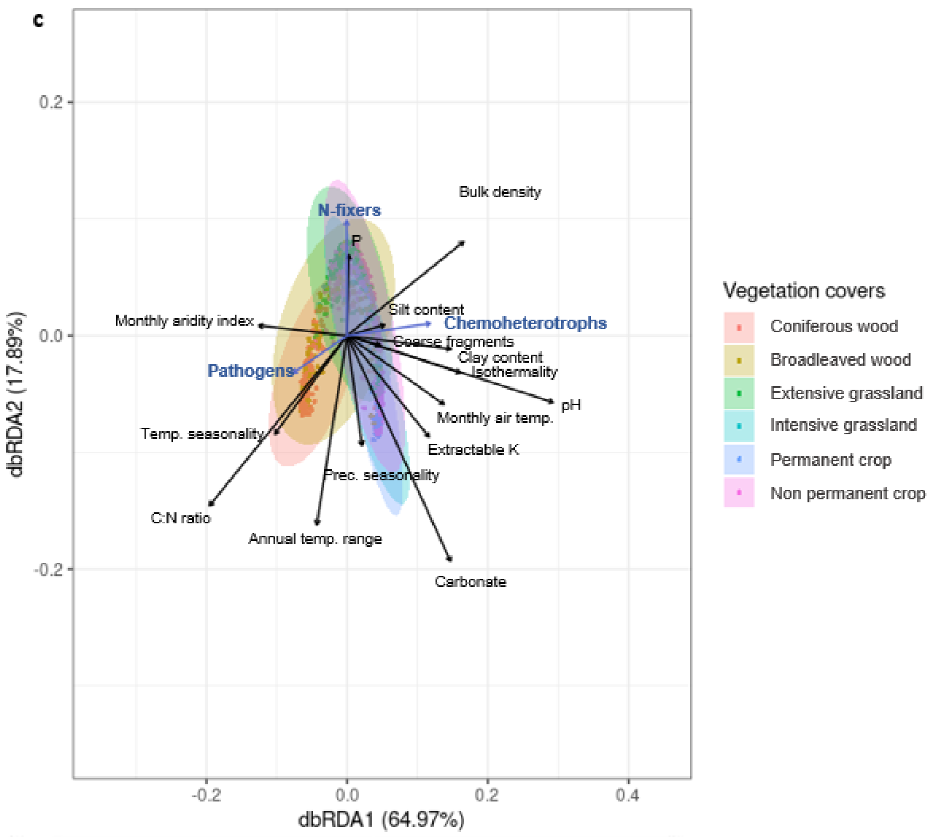
c
0.2
0.0
-0.2
-0.2
0.0
0.2
0.4
dbRDA2 (17.89%)
dbRDA1 (64.97%)
Bulk density
N-fixers
P
Silt content
Monthly aridity index
Chemoheterotrophs
Coarse fragments
Clay content
Isothermality
Pathogens
pH
Monthly air temp.
Temp. seasonality
Extractable K
Prec. seasonality
C:N ratio
Annual temp. range
Carbonate
Vegetation covers
Coniferous wood
Broadleaved wood
Extensive grassland
Intensive grassland
Permanent crop
Non permanent crop

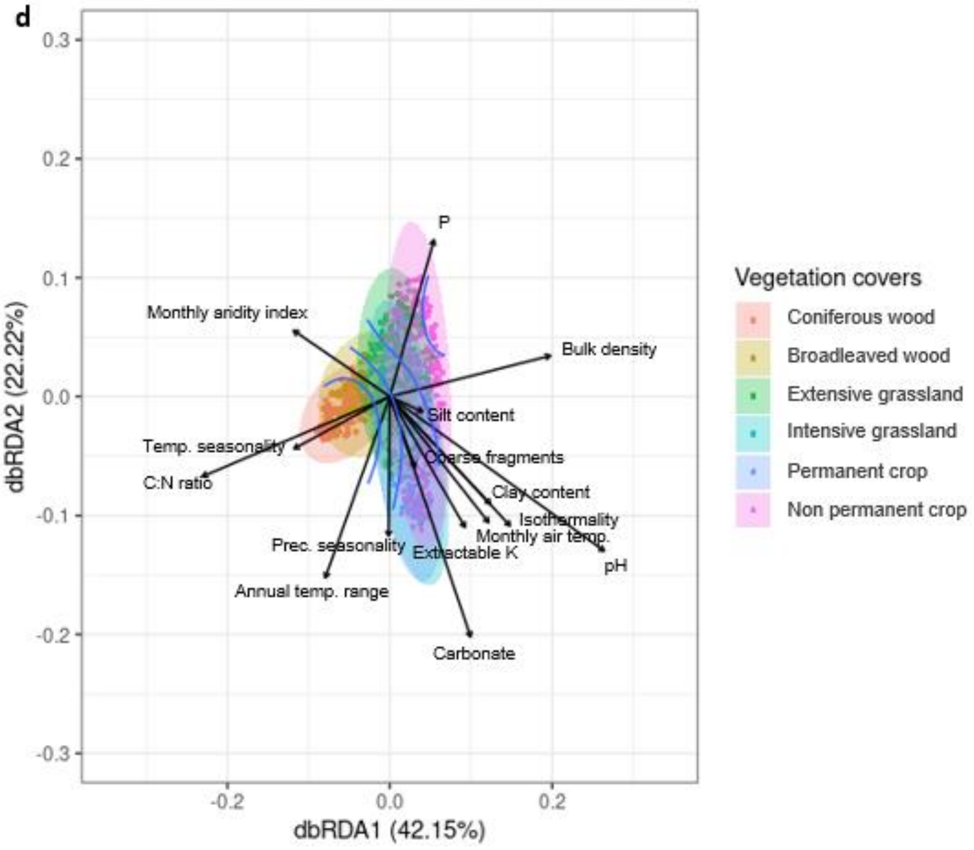
d
0.3
0.2
0.1
0.0
-0.1
-0.2
-0.3
-0.2
0.0
0.2
dbRDA2 (22.22%)
dbRDA1 (42.15%)
P
Monthly aridity index
Bulk density
Silt content
Temp. seasonality
Coarse fragments
C:N ratio
Clay content
Isothermality
Monthly air temp.
Prec. seasonality
Extractable K
pH
Annual temp. range
Carbonate
Vegetation covers
Coniferous wood
Broadleaved wood
Extensive grassland
Intensive grassland
Permanent crop
Non permanent crop

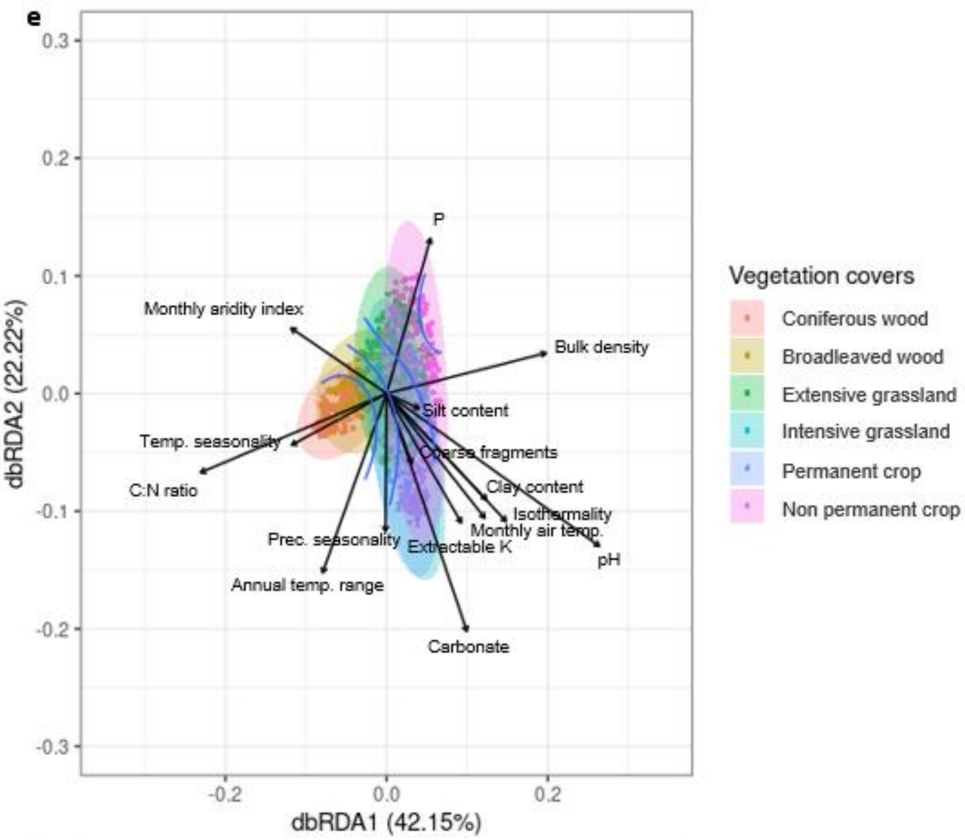
e
P
Monthly aridity index
Bulk density
Silt content
Temp. seasonality
Coarse fragments
C:N ratio
Clay content
Isothermality
Monthly air temp.
Prec. seasonality
Extractable K
pH
Annual temp. range
Carbonate
dbRDA2 (22.22%)
dbRDA1 (42.15%)
Vegetation covers
Coniferous wood
Broadleaved wood
Extensive grassland
Intensive grassland
Permanent crop
Non permanent crop

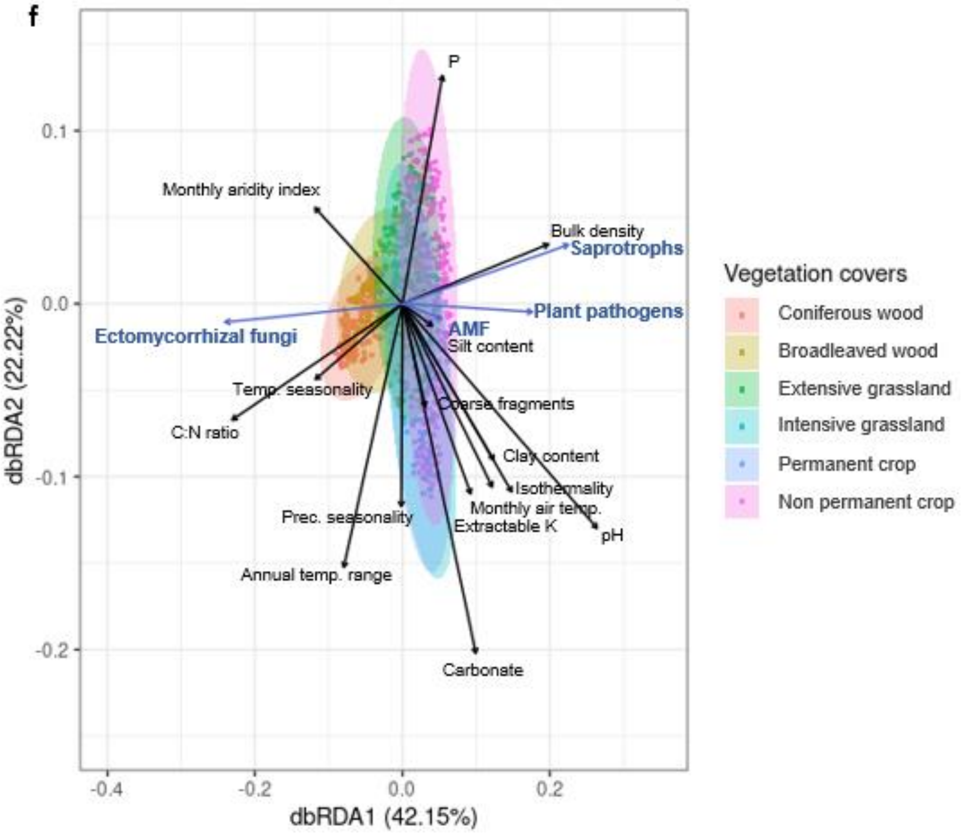
f
P
Monthly aridity index
Bulk density
Saprotrophs
Plant pathogens
Ectomycorrhizal fungi
AMF
Silt content
Temp. seasonality
Coarse fragments
C:N ratio
Clay content
Isothermality
Monthly air temp.
Prec. seasonality
Extractable K
pH
Annual temp. range
Carbonate
dbRDA2 (22.22%)
dbRDA1 (42.15%)
Vegetation covers
Coniferous wood
Broadleaved wood
Extensive grassland
Intensive grassland
Permanent crop
Non permanent crop

Supplementary Fig. 10.

Ordination biplots of the soil properties and climatic variables for bacteria and fungi (represented by black arrows). Points represent the sampling sites and are coloured by vegetation cover type. 95% confidence ellipses around groups of sites belonging to the same vegetation cover type are added and coloured accordingly. Isolines represent the values taken by **a** bacterial observed richness, **b** bacterial Shannon index, **d** fungal observed richness, **e** fungal Shannon index across the sites. Blue arrows represent the **c** bacterial and **f** fungal functional groups fitted onto the ordination. Source data are provided as a Source Data file.

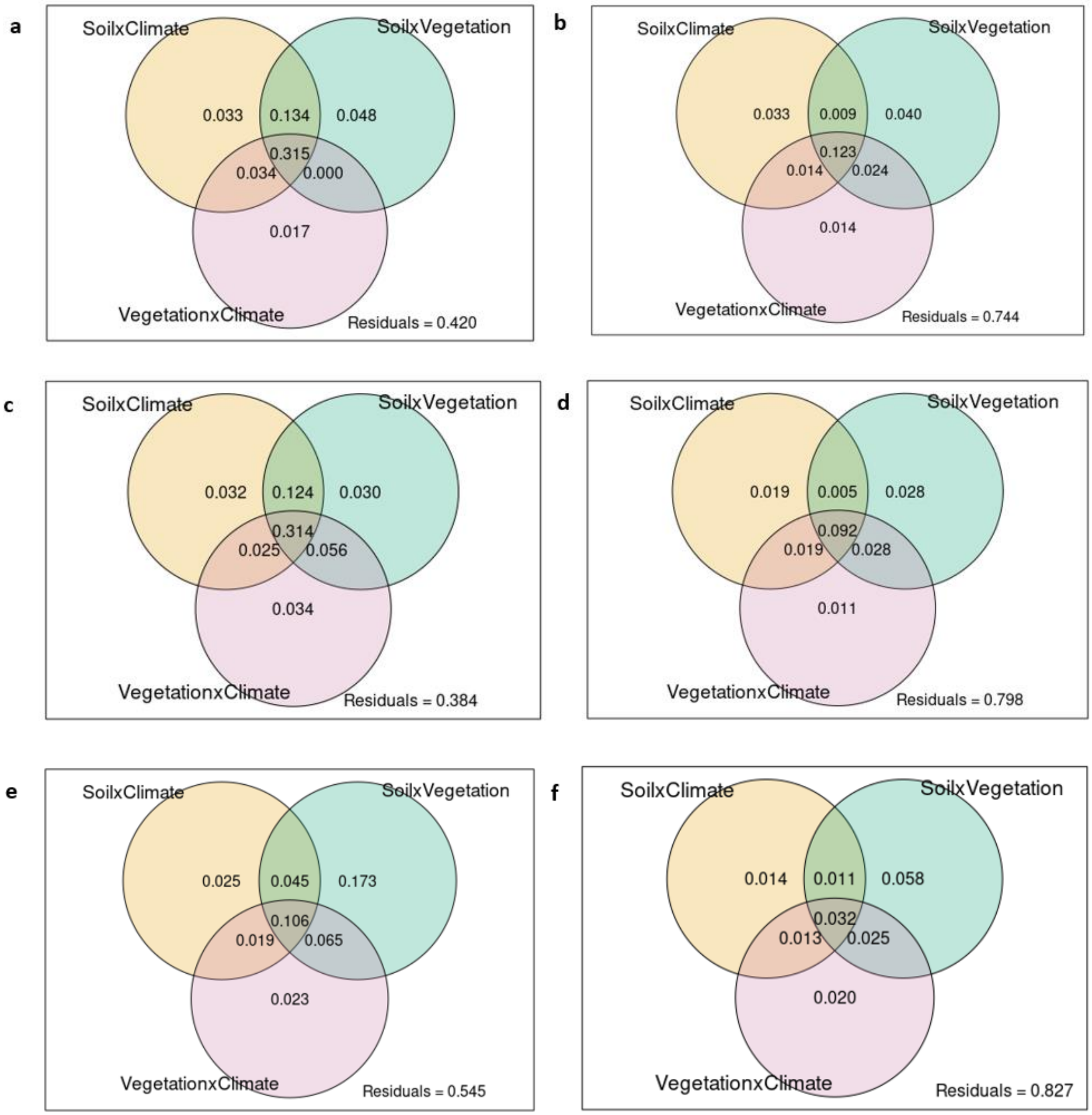

Supplementary Fig. 11.

Variation partitioning plots testing for the effect of the interactions between soil properties, vegetation cover and climate for **a** bacterial observed richness, **b** fungal observed richness, **c** bacterial Shannon index, **d** fungal Shannon index, **e** bacterial beta-diversity and **f** fungal beta-diversity. Here the interactions correspond to the interaction terms preselected in the models. Source data are provided as a Source Data file.

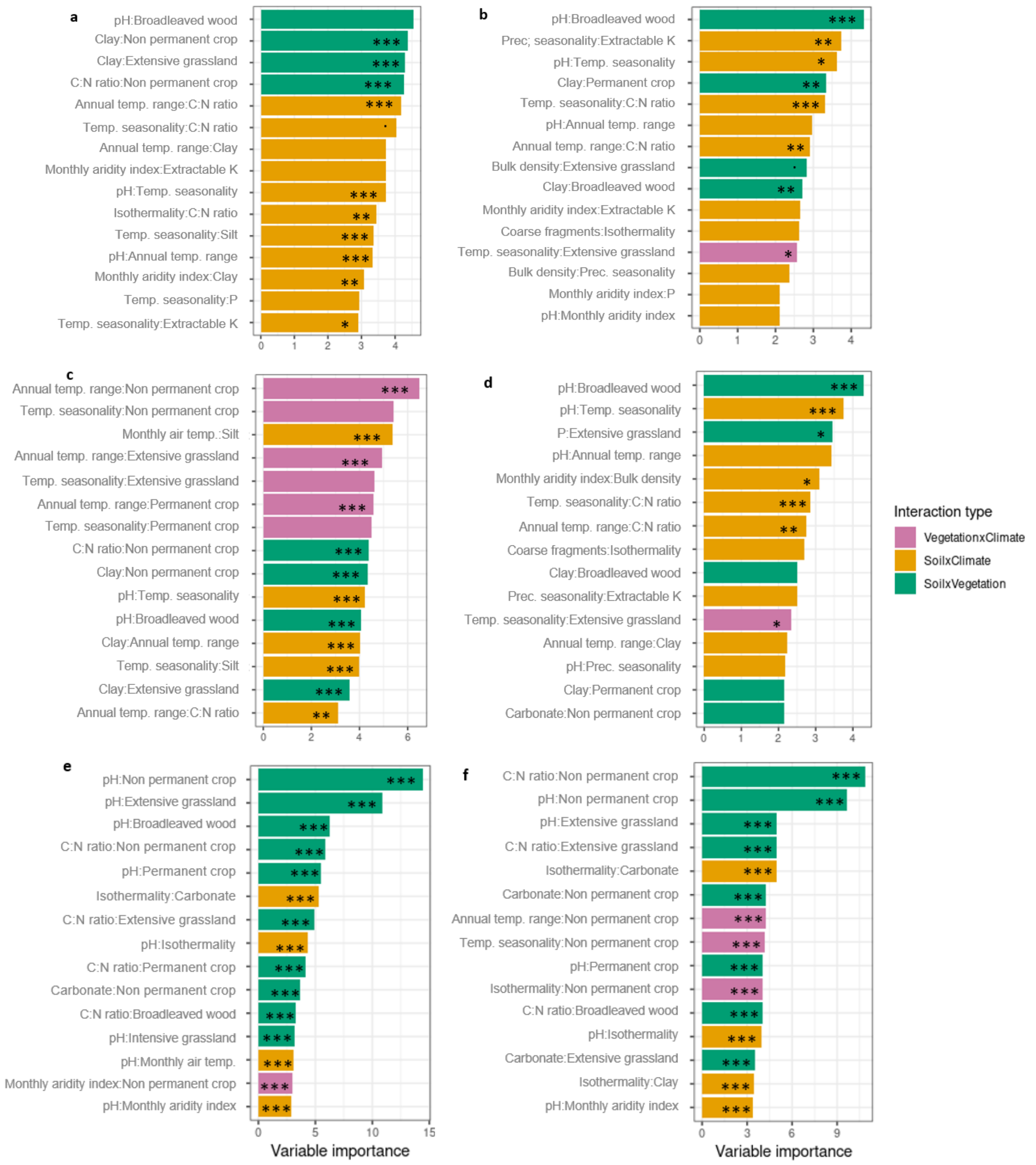

Supplementary Fig. 12.

Variable importance of the first fifteen two-way interactions in the multi-factorial models for **a** bacterial observed richness, **b** fungal observed richness, **c** bacterial Shannon index, **d** fungal Shnnon index, **e** bacterial beta-diversity and **f** fungal beta-diversity. Bars are coloured by interaction type. The stars represent the level of significance of the p-value for each term in the one-way ANOVA (**** $p < 0.001$; ** $p < 0.01$; * $p < 0.05$; . $p < 0.1$)*. Exact p-values are provided in Supplementary Data file 2. Source data are provided as a Source Data file.

a

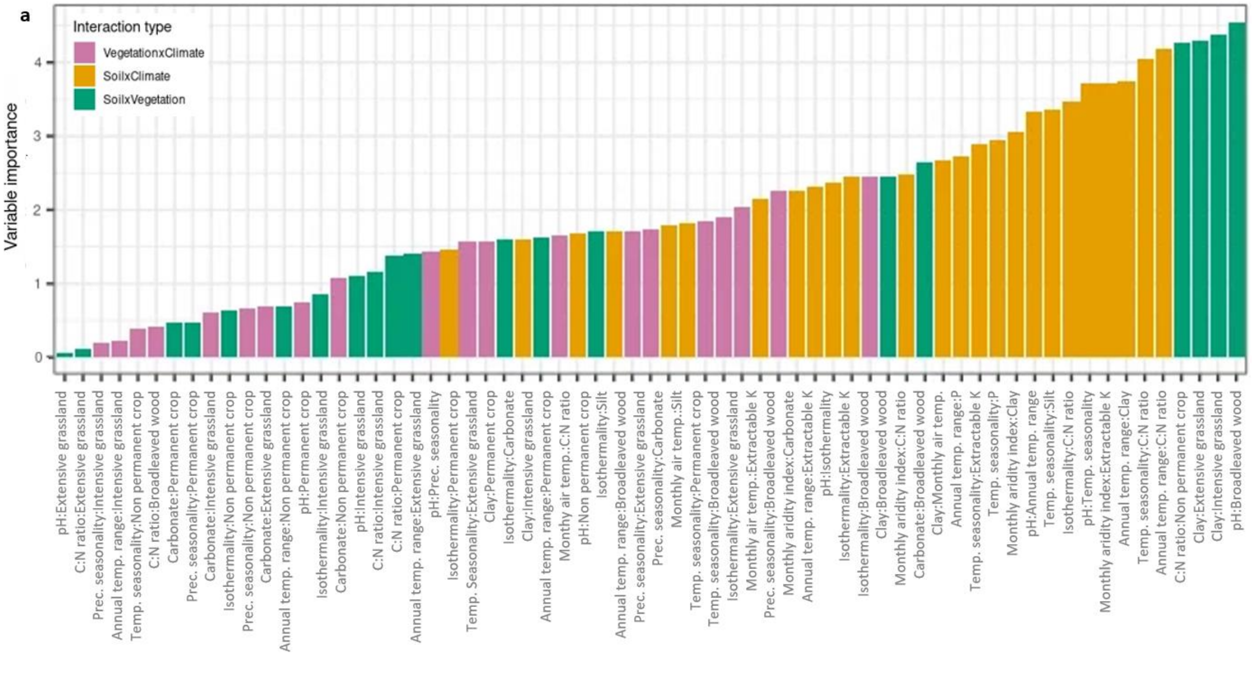
Interaction type
VegetationxClimate
SoilxClimate
SoilxVegetation
Variable importance
0
1
2
3
4
pH:Extensive grassland
C:N ratio:Extensive grassland
Prec. seasonality:Intensive grassland
Annual temp. range:Intensive grassland
Temp. seasonality:Non permanent crop
C:N ratio:Broadleaved wood
Carbonate:Permanent crop
Prec. seasonality:Permanent crop
Carbonate:Intensive grassland
Isothermality:Non permanent crop
Prec. seasonality:Non permanent crop
Carbonate:Extensive grassland
Annual temp. range:Non permanent crop
pH:Permanent crop
Isothermality:Intensive grassland
Carbonate:Non permanent crop
pH:Intensive grassland
C:N ratio:Intensive grassland
C:N ratio:Permanent crop
Annual temp. range:Extensive grassland
pH:Prec. seasonality
Isothermality:Permanent crop
Temp. Seasonality:Extensive grassland
Clay:Permanent crop
Isothermality:Carbonate
Clay:Intensive grassland
Annual temp. range:Permanent crop
Monthy air temp.:C:N ratio
pH:Non permanent crop
Isothermality:Silt
Annual temp. range:Broadleaved wood
Prec. seasonality:Extensive grassland
Prec. seasonality:Carbonate
Monthly air temp.:Silt
Temp. seasonality:Permanent crop
Temp. seasonality:Broadleaved wood
Isothermality:Extensive grassland
Monthly air temp.:Extractable K
Prec. seasonality:Broadleaved wood
Monthly aridity index:Carbonate
Annual temp. range:Extractable K
pH:Isothermality
Isothermality:Extractable K
Isothermality:Broadleaved wood
Clay:Broadleaved wood
Monthly aridity index:C:N ratio
Carbonate:Broadleaved wood
Clay:Monthly air temp.
Annual temp. range:P
Temp. seasonality:Extractable K
Temp. seasonality:P
Monthly aridity index:Clay
pH:Annual temp. range
Temp. seasonality:Silt
Isothermality:C:N ratio
pH:Temp. seasonality
Monthly aridity index:Extractable K
Annual temp. range:Clay
Temp. seasonality:C:N ratio
Annual temp. range:C:N ratio
C:N ratio:Non permanent crop
Clay:Extensive grassland
Clay:Intensive grassland
pH:Broadleaved wood

b

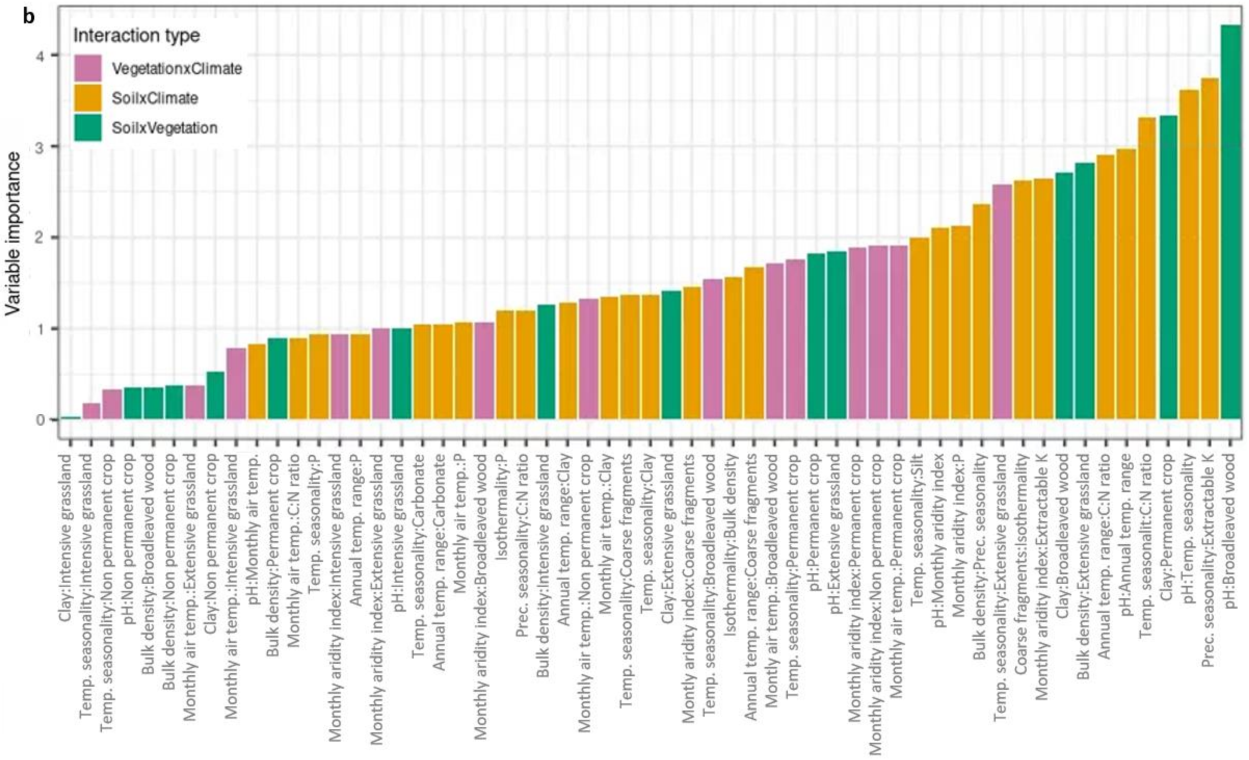
Interaction type
VegetationxClimate
SoilxClimate
SoilxVegetation
Variable importance
0
1
2
3
4
Clay:Intensive grassland
Temp. seasonality:Intensive grassland
Temp. seasonality:Non permanent crop
pH:Non permanent crop
Bulk density:Broadleaved wood
Bulk density:Non permanent crop
Monthly air temp.:Extensive grassland
Clay:Non permanent crop
Monthly air temp.:Intensive grassland
pH:Monthly air temp.
Bulk density:Permanent crop
Monthly air temp.:C:N ratio
Temp. seasonality:P
Monthly aridity index:Intensive grassland
Annual temp. range:P
Monthly aridity index:Extensive grassland
pH:Intensive grassland
Temp. seasonality:Carbonate
Annual temp. range:Carbonate
Monthly air temp.:P
Monthly aridity index:Broadleaved wood
Isothermality:P
Prec. seasonality:C:N ratio
Bulk density:Intensive grassland
Annual temp. range:Clay
Monthly air temp.:Non permanent crop
Monthly air temp.:Clay
Temp. seasonality:Coarse fragments
Temp. seasonality:Clay
Clay:Extensive grassland
Montly aridity index:Coarse fragments
Temp. seasonality:Broadleaved wood
Isothermality:Bulk density
Annual temp. range:Coarse fragments
Montly air temp.:Broadleaved wood
Temp. seasonality:Permanent crop
pH:Permanent crop
pH:Extensive grassland
Monthly aridity index:Permanent crop
Monthly aridity index:Non permanent crop
Monthly air temp.:Permanent crop
Temp. seasonality:Silt
pH:Monthly aridity index
Monthly aridity index:P
Bulk density:Prec. seasonality
Temp. seasonality:Extensive grassland
Coarse fragments:Isothermality
Monthly aridity index:Extractable K
Clay:Broadleaved wood
Bulk density:Extensive grassland
Annual temp. range:C:N ratio
pH:Annual temp. range
Temp. seasonalit:C:N ratio
Clay:Permanent crop
pH:Temp. seasonality
Prec. seasonality:Extractable K
pH:Broadleaved wood

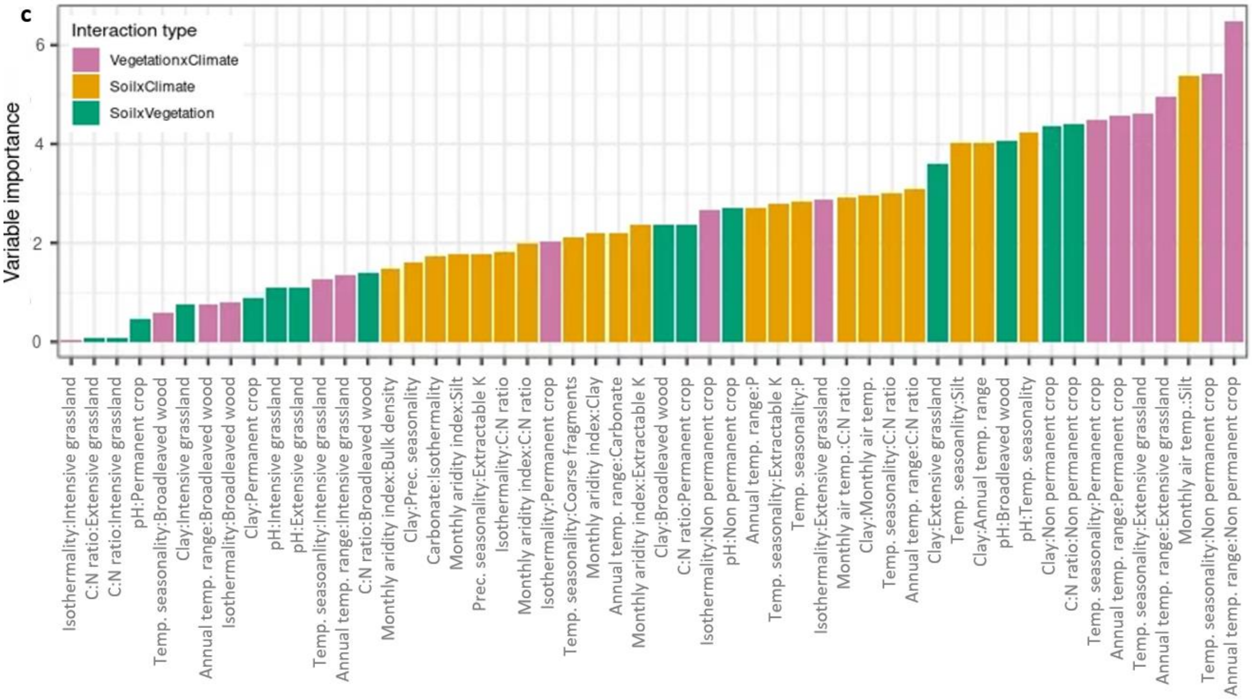
c
Interaction type
VegetationxClimate
SoilxClimate
SoilxVegetation
Variable importance
0
2
4
6
Isothermality:Intensive grassland
C:N ratio:Extensive grassland
C:N ratio:Intensive grassland
pH:Permanent crop
Temp. seasonality:Broadleaved wood
Clay:Intensive grassland
Annual temp. range:Broadleaved wood
Isothermality:Broadleaved wood
Clay:Permanent crop
pH:Intensive grassland
pH:Extensive grassland
Temp. seasoanlity:Intensive grassland
Annual temp. range:Intensive grassland
C:N ratio:Broadleaved wood
Monthly aridity index:Bulk density
Clay:Prec. seasonality
Carbonate:Isothermality
Monthly aridity index:Silt
Prec. seasonality:Extractable K
Isothermality:C:N ratio
Monthly aridity index:C:N ratio
Isothermality:Permanent crop
Temp. seasonality:Coarse fragments
Monthly aridity index:Clay
Annual temp. range:Carbonate
Monthly aridity index:Extractable K
Clay:Broadleaved wood
C:N ratio:Permanent crop
Isothermality:Non permanent crop
pH:Non permanent crop
Annual temp. range:P
Temp. seasonality:Extractable K
Temp. seasonality:P
Isothermality:Extensive grassland
Monthly air temp.:C:N ratio
Clay:Monthly air temp.
Temp. seasonality:C:N ratio
Annual temp. range:C:N ratio
Clay:Extensive grassland
Temp. seasoanlity:Silt
Clay:Annual temp. range
pH:Broadleaved wood
pH:Temp. seasonality
Clay:Non permanent crop
C:N ratio:Non permanent crop
Temp. seasonality:Permanent crop
Annual temp. range:Permanent crop
Temp. seasonality:Extensive grassland
Annual temp. range:Extensive grassland
Monthly air temp.:Silt
Temp. seasonality:Non permanent crop
Annual temp. range:Non permanent crop

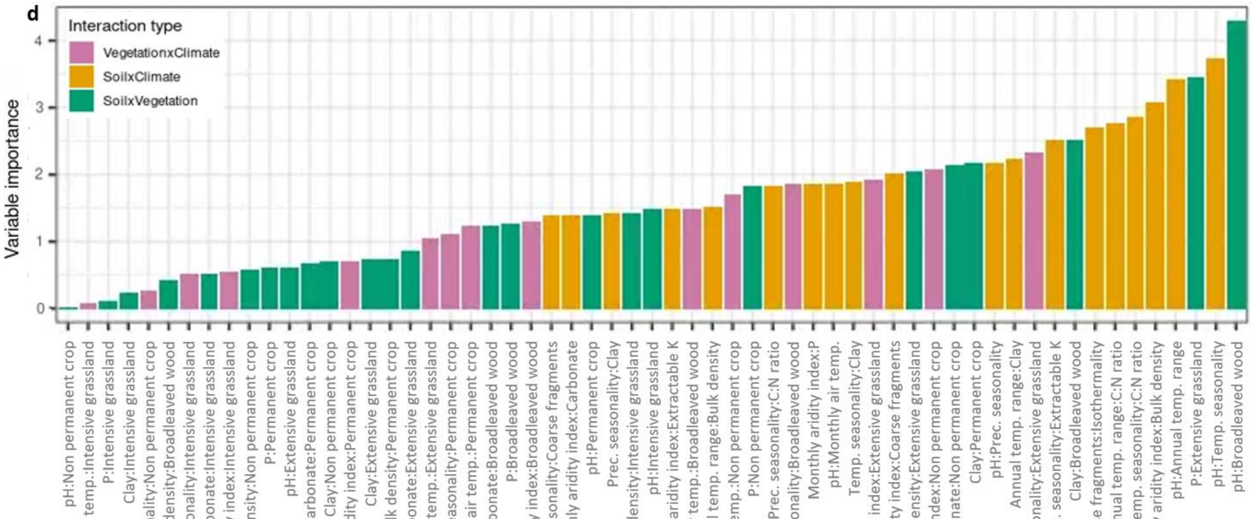
d
Interaction type
VegetationxClimate
SoilxClimate
SoilxVegetation
Variable importance
0
1
2
3
4
pH:Non permanent crop
Monthly air temp.:Intensive grassland
P:Intensive grassland
Clay:Intensive grassland
Temp. seasonality:Non permanent crop
Bulk density:Broadleaved wood
Temp. seasonality:Intensive grassland
Carbonate:Intensive grassland
Monthly aridity index:Intensive grassland
Bulk density:Non permanent crop
P:Permanent crop
pH:Extensive grassland
Carbonate:Permanent crop
Clay:Non permanent crop
Monthly aridity index:Permanent crop
Clay:Extensive grassland
Bulk density:Permanent crop
Carbonate:Extensive grassland
Monthly air temp.:Extensive grassland
Temp. seasonality:Permanent crop
Monthly air temp.:Permanent crop
Carbonate:Broadleaved wood
P:Broadleaved wood
Monthly aridity index:Broadleaved wood
Prec. seasonality:Coarse fragments
Monthly aridity index:Carbonate
pH:Permanent crop
Prec. seasonality:Clay
Bulk density:Intensive grassland
pH:Intensive grassland
Monthly aridity index:Extractable K
Monthly air temp.:Broadleaved wood
Annual temp. range:Bulk density
Monthly air temp.:Non permanent crop
P:Non permanent crop
Prec. seasonality:C:N ratio
Temp. seasonality:Broadleaved wood
Monthly aridity index:P
pH:Monthly air temp.
Temp. seasonality:Clay
Monthly aridity index:Extensive grassland
Monthly aridity index:Coarse fragments
Bulk density:Extensive grassland
Monthly aridity index:Non permanent crop
Carbonate:Non permanent crop
Clay:Permanent crop
pH:Prec. seasonality
Annual temp. range:Clay
Temp. seasonality:Extensive grassland
Prec. seasonality:Extractable K
Clay:Broadleaved wood
Coarse fragments:Isothermality
Annual temp. range:C:N ratio
Temp. seasonality:C:N ratio
Monthly aridity index:Bulk density
pH:Annual temp. range
P:Extensive grassland
pH:Temp. seasonality
pH:Broadleaved wood

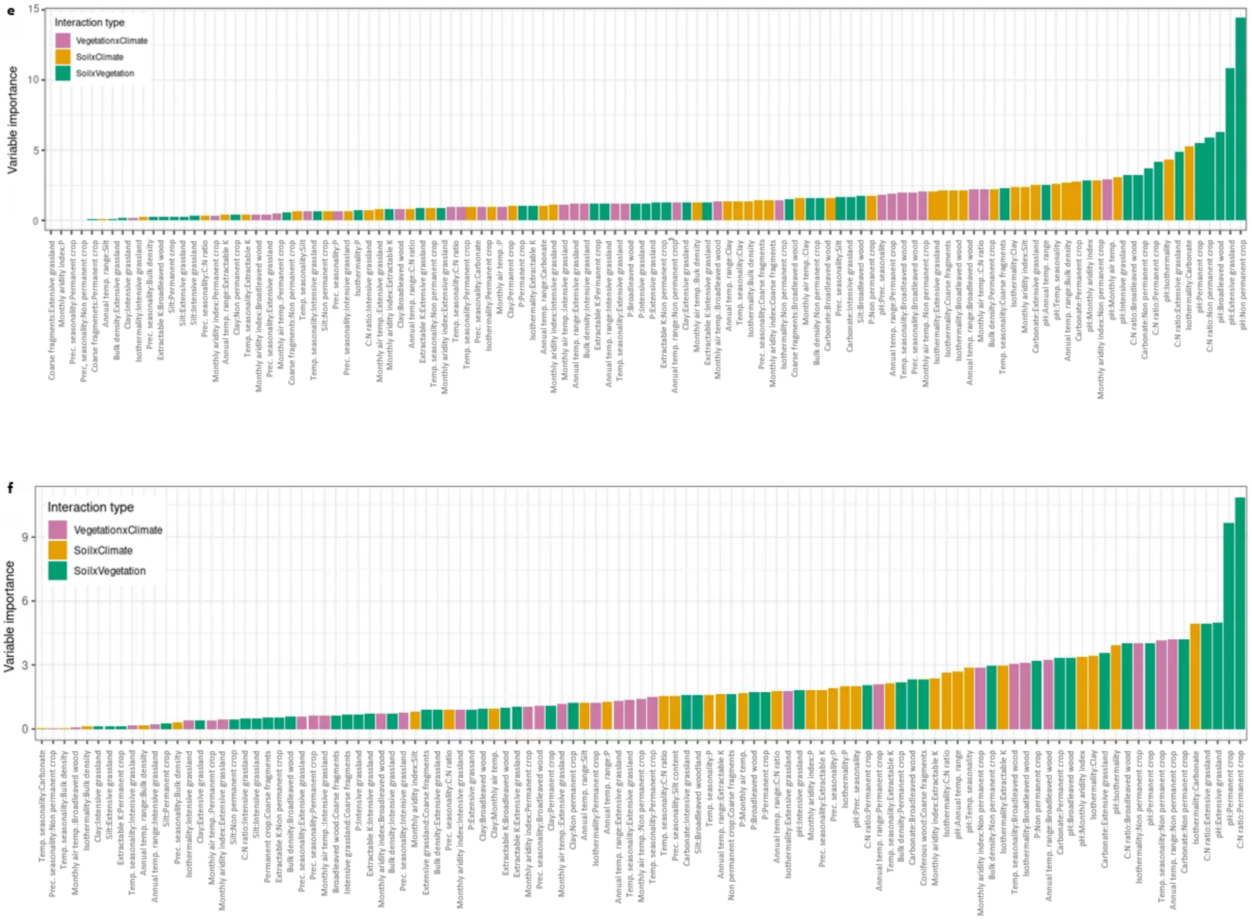

Supplementary Fig. 13.

Complete barplots of the variable importance of the interaction terms preselected in the interaction models for **a** bacterial observed richness, **b** fungal observed richness, **c** bacterial Shannon index, **d** fungal Shannon index, **e** bacterial beta-diversity and **f** fungal beta-diversity (Bray-Curtis dissimilarity matrix calculated on the Hellinger-transformed sample-by-(z)OTU table). Bars are coloured by interaction type. Source data are provided as a Source Data file.

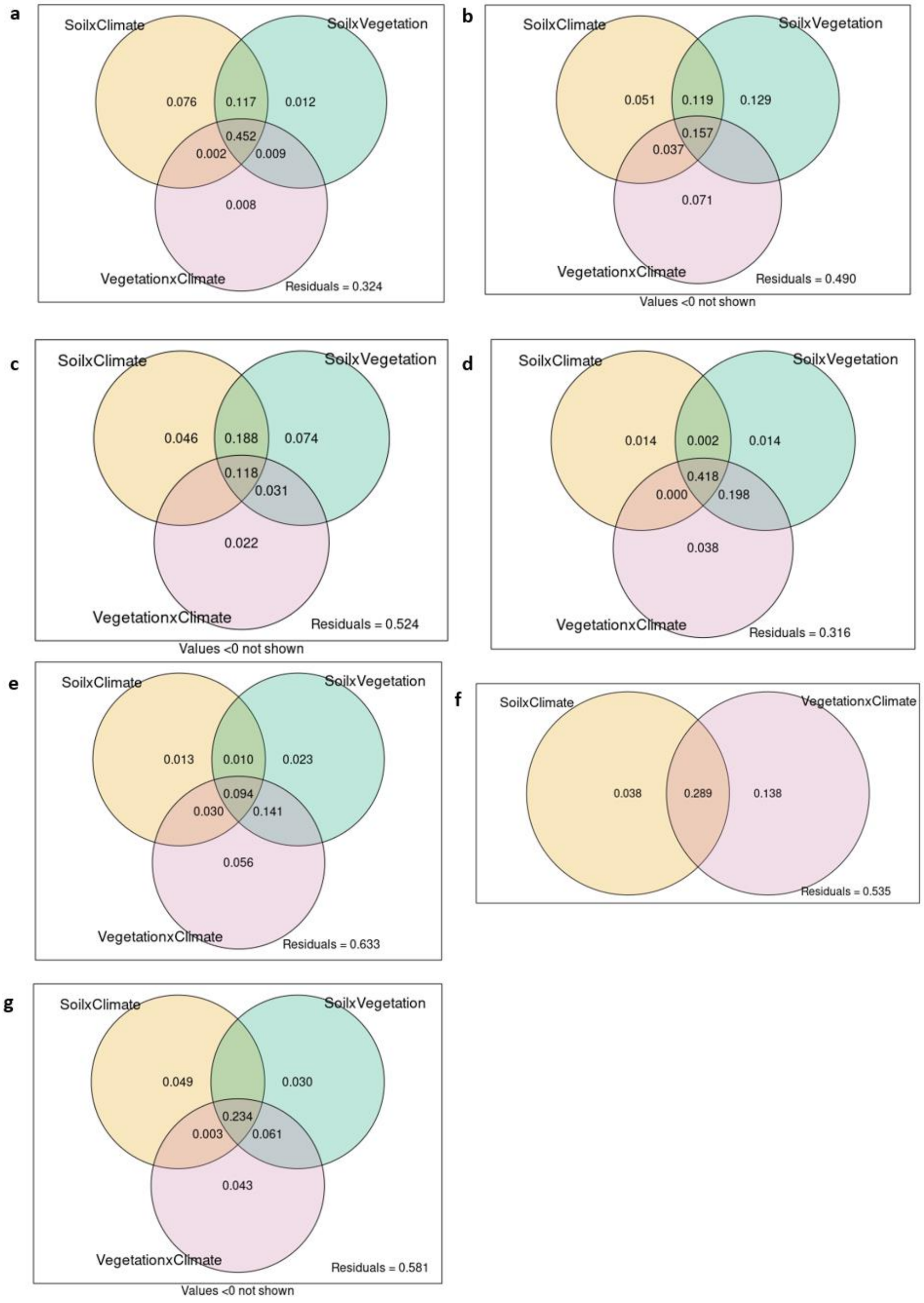

Supplementary Fig. 14.

Variation partitioning plots testing for the effect of the interactions between soil properties, vegetation cover and climate on bacterial and fungal functional groups for **a** bacterial chemoheterotrophs, **b** bacterial N-fixers, **c** bacterial pathogens, **d** ectomycorrhizal fungi, **e** arbuscular mycorrhizal fungi, **f** fungal saprotrophs and **g** fungal plant pathogens. Here the interactions correspond to the interaction terms preselected in the models. Source data are provided as a Source Data file.

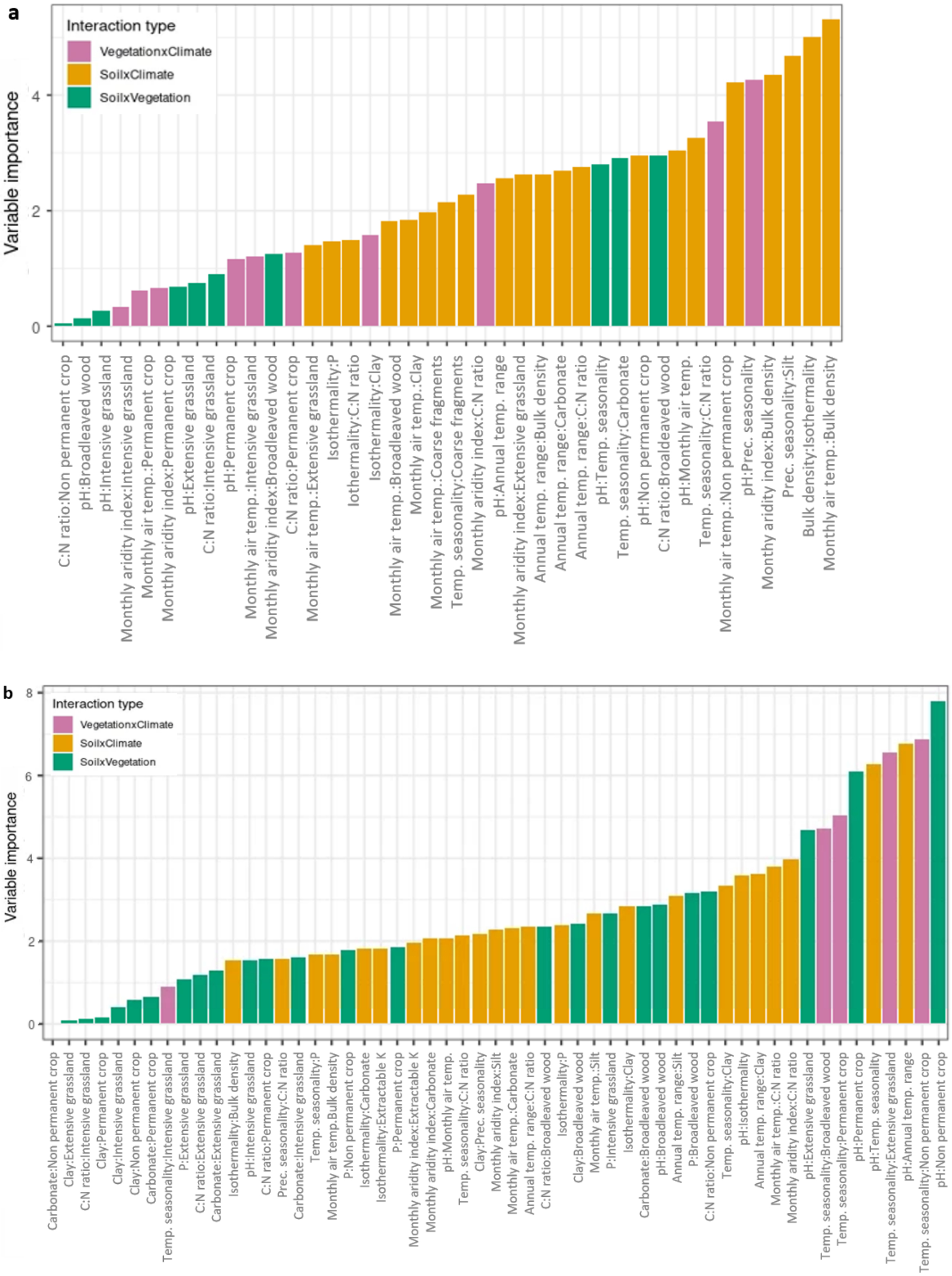
a
Interaction type
VegetationxClimate
SoilxClimate
SoilxVegetation
Variable importance
0
2
4
C:N ratio:Non permanent crop
pH:Broadleaved wood
pH:Intensive grassland
Monthly aridity index:Intensive grassland
Monthly air temp.:Permanent crop
Monthly aridity index:Permanent crop
pH:Extensive grassland
C:N ratio:Intensive grassland
pH:Permanent crop
Monthly air temp.:Intensive grassland
Monthly aridity index:Broadleaved wood
C:N ratio:Permanent crop
Monthly air temp.:Extensive grassland
Isothermality:P
Iothermality:C:N ratio
Isothermality:Clay
Monthly air temp.:Broadleaved wood
Monthly air temp.:Clay
Monthly air temp.:Coarse fragments
Temp. seasonality:Coarse fragments
Monthly aridity index:C:N ratio
pH:Annual temp. range
Monthly aridity index:Extensive grassland
Annual temp. range:Bulk density
Annual temp. range:Carbonate
Annual temp. range:C:N ratio
pH:Temp. seasonality
Temp. seasonality:Carbonate
pH:Non permanent crop
C:N ratio:Broadleaved wood
pH:Monthly air temp.
Temp. seasonality:C:N ratio
Monthly air temp.:Non permanent crop
pH:Prec. seasonality
Monthy aridity index:Bulk density
Prec. seasonality:Silt
Bulk density:Isothermality
Monthly air temp.:Bulk density
b
Interaction type
VegetationxClimate
SoilxClimate
SoilxVegetation
Variable importance
0
2
4
6
8
Carbonate:Non permanent crop
Clay:Extensive grassland
C:N ratio:Intensive grassland
Clay:Permanent crop
Clay:Intensive grassland
Clay:Non permanent crop
Carbonate:Permanent crop
Temp. seasonality:Intensive grassland
P:Extensive grassland
C:N ratio:Extensive grassland
Carbonate:Extensive grassland
Isothermality:Bulk density
pH:Intensive grassland
C:N ratio:Permanent crop
Prec. seasonality:C:N ratio
Carbonate:Intensive grassland
Temp. seasonality:P
Monthly air temp.Bulk density
P:Non permanent crop
Isothermality:Carbonate
Isothermality:Extractable K
P:Permanent crop
Monthly aridity index:Extractable K
Monthly aridity index:Carbonate
pH:Monthly air temp.
Temp. seasonality:C:N ratio
Clay:Prec. seasonality
Monthly aridity index:Silt
Monthly air temp.:Carbonate
Annual temp. range:C:N ratio
C:N ratio:Broadleaved wood
Isothermality:P
Clay:Broadleaved wood
Monthly air temp.:Silt
P:Intensive grassland
Isothermality:Clay
Carbonate:Broadleaved wood
pH:Broadleaved wood
Annual temp. range:Silt
P:Broadleaved wood
C:N ratio:Non permanent crop
Temp. seasonality:Clay
pH:Isothermality
Annual temp. range:Clay
Monthly air temp.:C:N ratio
Monthly aridty index:C:N ratio
pH:Extensive grassland
Temp. seasonality:Broadleaved wood
Temp. seasonality:Permanent crop
pH:Permanent crop
pH:Temp. seasonality
Temp. seasonality:Extensive grassland
pH:Annual temp. range
Temp. seasonality:Non permanent crop
pH:Non permanent crop

c

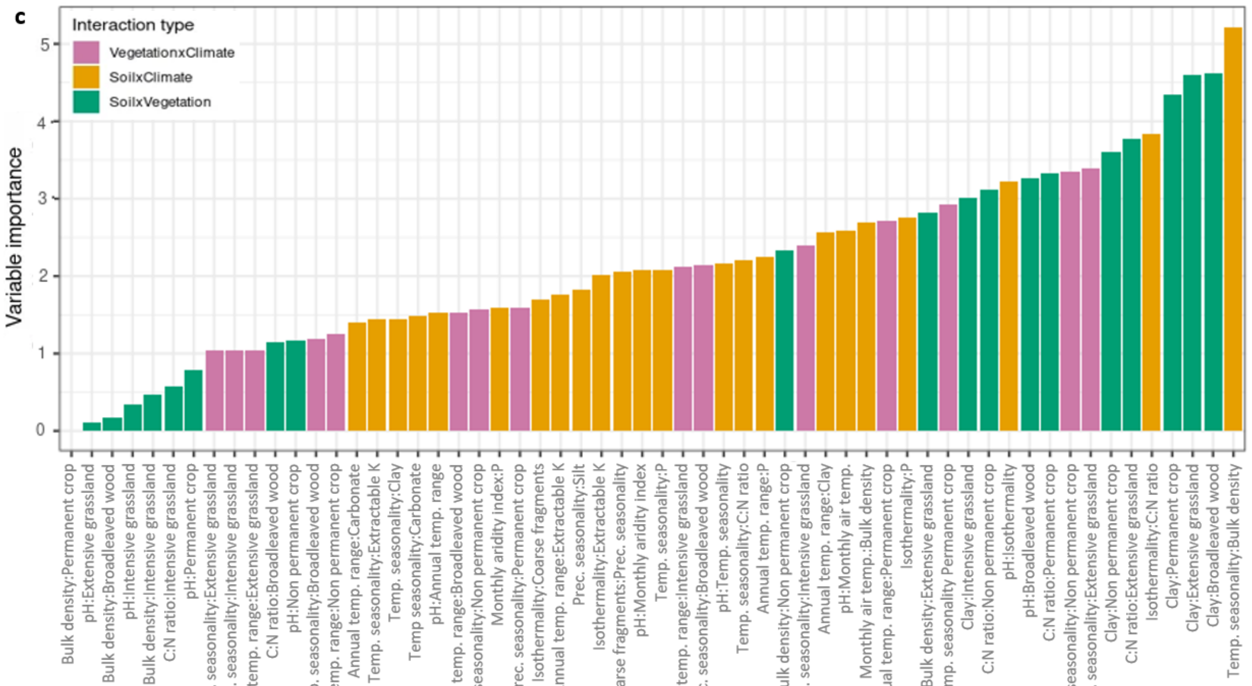

Interaction type
VegetationxClimate
SoilxClimate
SoilxVegetation
Variable importance
0
1
2
3
4
5
Bulk density:Permanent crop
pH:Extensive grassland
Bulk density:Broadleaved wood
pH:Intensive grassland
Bulk density:Intensive grassland
C:N ratio:Intensive grassland
pH:Permanent crop
Temp. seasonality:Extensive grassland
Prec. seasonality:Intensive grassland
Annual temp. range:Extensive grassland
C:N ratio:Broadleaved wood
pH:Non permanent crop
Temp. seasonality:Broadleaved wood
Annual temp. range:Non permanent crop
Annual temp. range:Carbonate
Temp. seasonality:Extractable K
Temp. seasonality:Clay
Temp seasonality:Carbonate
pH:Annual temp. range
Annual temp. range:Broadleaved wood
Temp. seasonality:Non permanent crop
Monthly aridity index:P
Prec. seasonality:Permanent crop
Isothermality:Coarse fragments
Annual temp. range:Extractable K
Prec. seasonality:Silt
Isothermality:Extractable K
Coarse fragments:Prec. seasonality
pH:Monthly aridity index
Temp. seasonality:P
Annual temp. range:Intensive grassland
Prec. seasonality:Broadleaved wood
pH:Temp. seasonality
Temp. seasonality:C:N ratio
Annual temp. range:P
Bulk density:Non permanent crop
Temp. seasonality:Intensive grassland
Annual temp. range:Clay
pH:Monthly air temp.
Monthly air temp.:Bulk density
Annual temp. range:Permanent crop
Isothermality:P
Bulk density:Extensive grassland
Temp. seasonality Permanent crop
Clay:Intensive grassland
C:N ratio:Non permanent crop
pH:Isothermality
pH:Broadleaved wood
C:N ratio:Permanent crop
Prec. seasonality:Non permanent crop
Prec. seasonality:Extensive grassland
Clay:Non permanent crop
C:N ratio:Extensive grassland
Isothermality:C:N ratio
Clay:Permanent crop
Clay:Extensive grassland
Clay:Broadleaved wood
Temp. seasonality:Bulk density

d

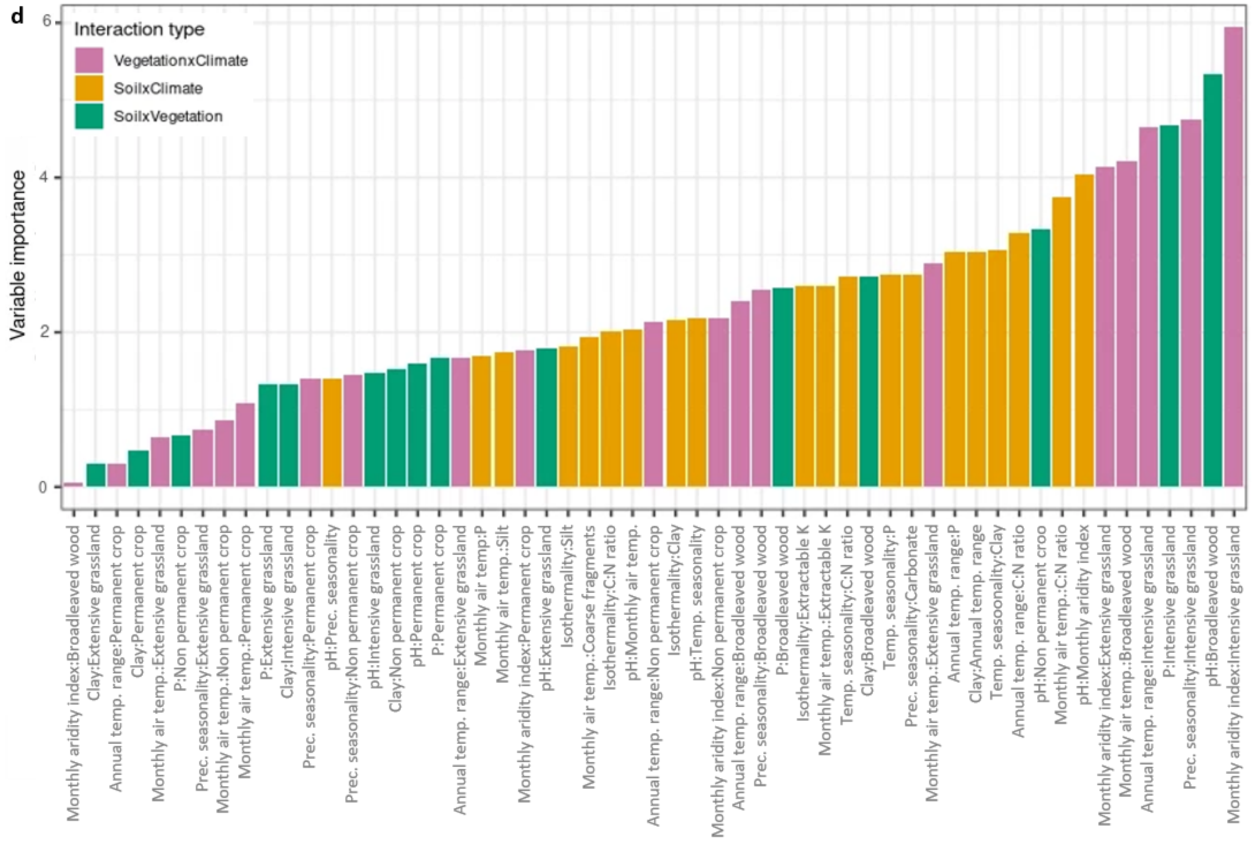

Interaction type
VegetationxClimate
SoilxClimate
SoilxVegetation
Variable importance
0
2
4
6
Monthly aridity index:Broadleaved wood
Clay:Extensive grassland
Annual temp. range:Permanent crop
Clay:Permanent crop
Monthly air temp.:Extensive grassland
P:Non permanent crop
Prec. seasonality:Extensive grassland
Monthly air temp.:Non permanent crop
Monthly air temp.:Permanent crop
P:Extensive grassland
Clay:Intensive grassland
Prec. seasonality:Permanent crop
pH:Prec. seasonality
Prec. seasonality:Non permanent crop
pH:Intensive grassland
Clay:Non permanent crop
pH:Permanent crop
P:Permanent crop
Annual temp. range:Extensive grassland
Monthly air temp:P
Monthly air temp.:Silt
Monthly aridity index:Permanent crop
pH:Extensive grassland
Isothermality:Silt
Monthly air temp.:Coarse fragments
Isothermality:C:N ratio
pH:Monthly air temp.
Annual temp. range:Non permanent crop
Isothermality:Clay
pH:Temp. seasonality
Monthly aridity index:Non permanent crop
Annual temp. range:Broadleaved wood
Prec. seasonality:Broadleaved wood
P:Broadleaved wood
Isothermality:Extractable K
Monthly air temp.:Extractable K
Temp. seasonality:C:N ratio
Clay:Broadleaved wood
Temp. seasonality:P
Prec. seasonality:Carbonate
Monthly air temp.:Extensive grassland
Annual temp. range:P
Clay:Annual temp. range
Temp. seasoonality:Clay
Annual temp. range:C:N ratio
pH:Non permanent croo
Monthly air temp.:C:N ratio
pH:Monthly aridity index
Monthly aridity index:Extensive grassland
Monthly air temp.:Broadleaved wood
Annual temp. range:Intensive grassland
P:Intensive grassland
Prec. seasonality:Intensive grassland
pH:Broadleaved wood
Monthly aridity index:Intensive grassland

e

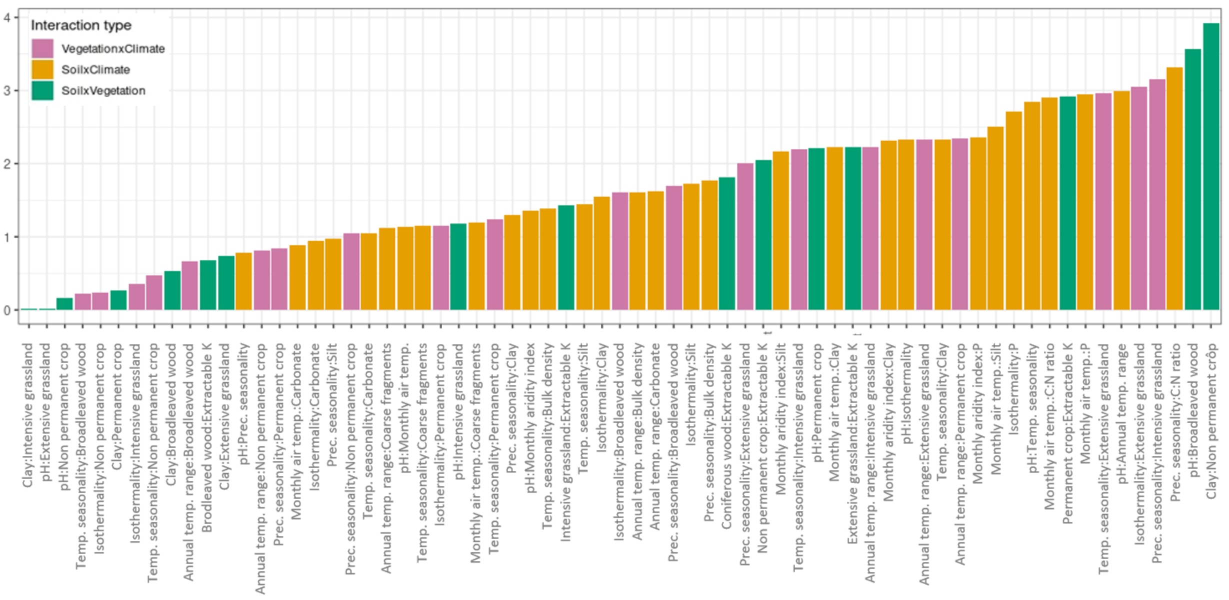

Interaction type
VegetationxClimate
SoilxClimate
SoilxVegetation
Variable importance
0
1
2
3
4
Clay:Intensive grassland
pH:Extensive grassland
pH:Non permanent crop
Temp. seasonality:Broadleaved wood
Isothermality:Non permanent crop
Clay:Permanent crop
Isothermality:Intensive grassland
Temp. seasonality:Non permanent crop
Clay:Broadleaved wood
Annual temp. range:Broadleaved wood
Brodleaved wood:Extractable K
Clay:Extensive grassland
pH:Prec. seasonality
Annual temp. range:Non permanent crop
Prec. seasonality:Permanent crop
Monthly air temp.:Carbonate
Isothermality:Carbonate
Prec. seasonality:Silt
Prec. seasonality:Non permanent crop
Temp. seasonality:Carbonate
Annual temp. range:Coarse fragments
pH:Monthly air temp.
Temp. seasonality:Coarse fragments
Isothermality:Permanent crop
pH:Intensive grassland
Monthly air temp.:Coarse fragments
Temp. seasonality:Permanent crop
Prec. seasonality:Clay
pH:Monthly aridity index
Temp. seasonality:Bulk density
Intensive grassland:Extractable K
Temp. seasonality:Silt
Isothermality:Clay
Isothermality:Broadleaved wood
Annual temp. range:Bulk density
Annual temp. range:Carbonate
Prec. seasonality:Broadleaved wood
Isothermality:Silt
Prec. seasonality:Bulk density
Coniferous wood:Extractable K
Prec. seasonality:Extensive grassland
Non permanent crop:Extractable K
Monthly aridity index:Silt
Temp. seasonality:Intensive grassland
pH:Permanent crop
Monthly air temp.:Clay
Extensive grassland:Extractable K
Annual temp. range:Intensive grassland
Monthly aridity index:Clay
pH:Isothermality
Annual temp. range:Extensive grassland
Temp. seasonality:Clay
Annual temp. range:Permanent crop
Monthly aridity index:P
Monthly air temp.:Silt
Isothermality:P
pH:Temp. seasonality
Monthly air temp.:C:N ratio
Permanent crop:Extractable K
Monthly air temp.:P
Temp. seasonality:Extensive grassland
pH:Annual temp. range
Isothermality:Extensive grassland
Prec. seasonality:Intensive grassland
Prec. seasonality:C:N ratio
pH:Broadleaved wood
Clay:Non permanent crop

f

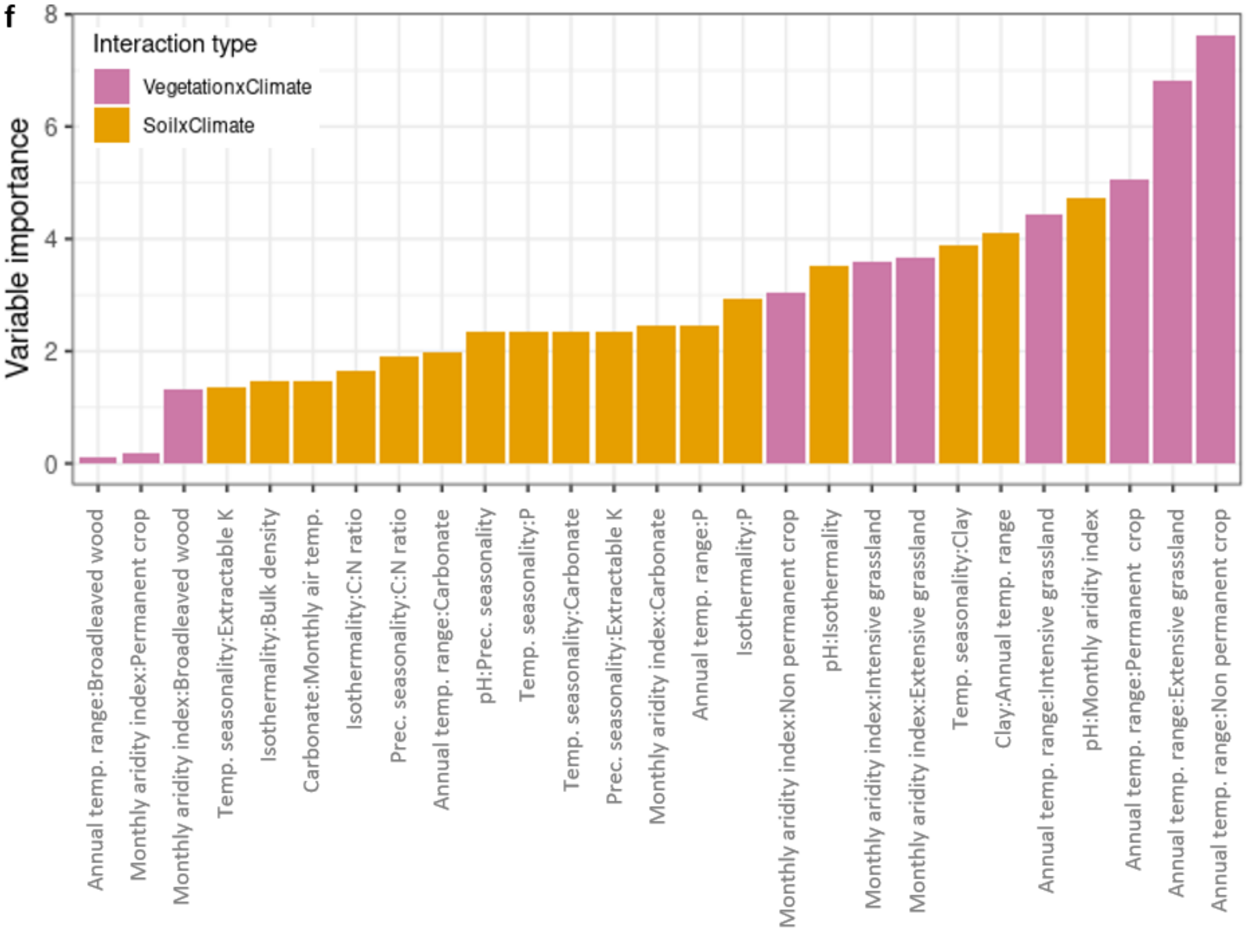

Interaction type
VegetationxClimate
SoilxClimate
Variable importance
0
2
4
6
8
Annual temp. range:Broadleaved wood
Monthly aridity index:Permanent crop
Monthly aridity index:Broadleaved wood
Temp. seasonality:Extractable K
Isothermality:Bulk density
Carbonate:Monthly air temp.
Isothermality:C:N ratio
Prec. seasonality:C:N ratio
Annual temp. range:Carbonate
pH:Prec. seasonality
Temp. seasonality:P
Temp. seasonality:Carbonate
Prec. seasonality:Extractable K
Monthly aridity index:Carbonate
Annual temp. range:P
Isothermality:P
Monthly aridity index:Non permanent crop
pH:Isothermality
Monthly aridity index:Intensive grassland
Monthly aridity index:Extensive grassland
Temp. seasonality:Clay
Clay:Annual temp. range
Annual temp. range:Intensive grassland
pH:Monthly aridity index
Annual temp. range:Permanent crop
Annual temp. range:Extensive grassland
Annual temp. range:Non permanent crop

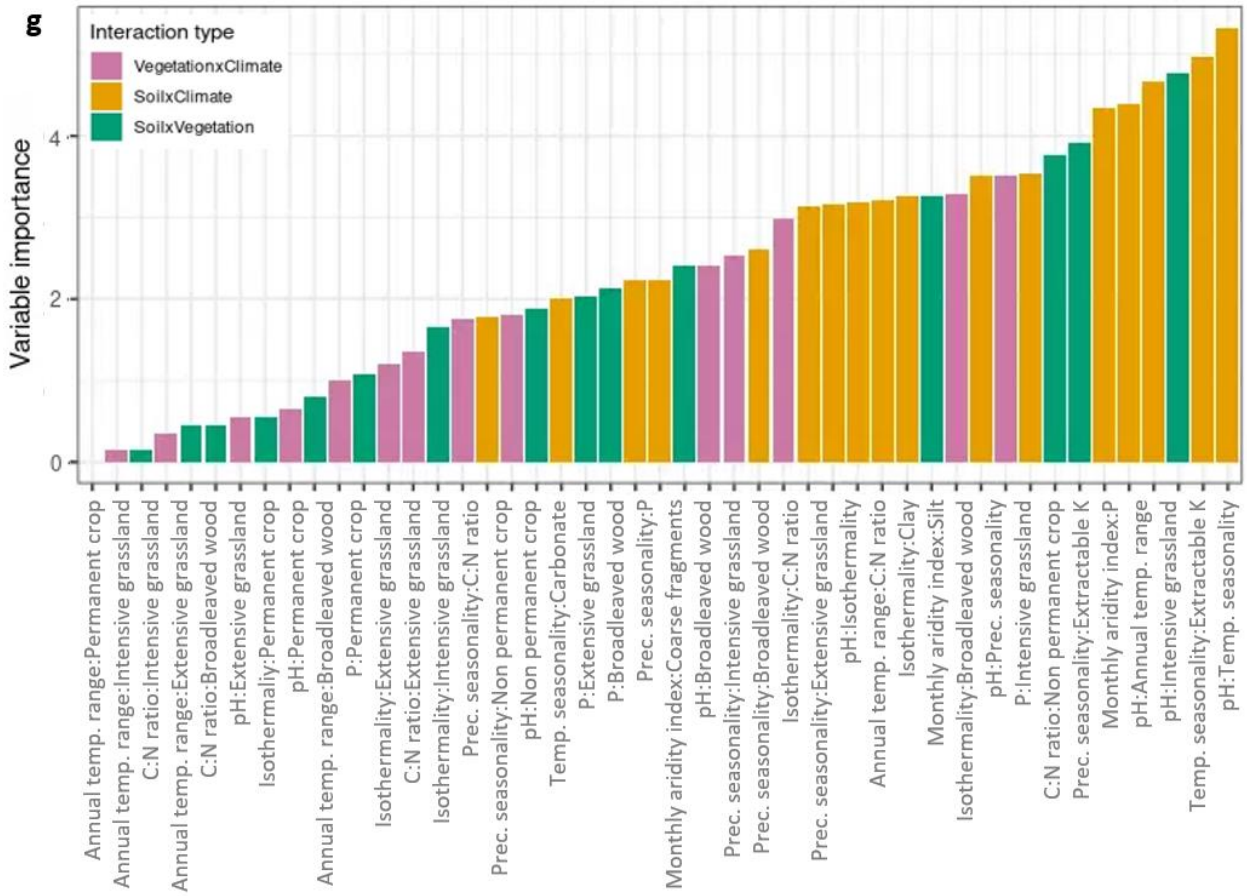

Supplementary Fig. 15.

Complete barplots of the variable importance of the interaction terms preselected in the interaction models **a** bacterial chemoheterotrophs, **b** bacterial N-fixers, **c** bacterial pathogens, **d** ectomycorrhizal fungi, **e** arbuscular mycorrhizal fungi, **f** fungal saprotrophs and **g** fungal plant pathogens. Bars are coloured by interaction type. Source data are provided as a Source Data file.

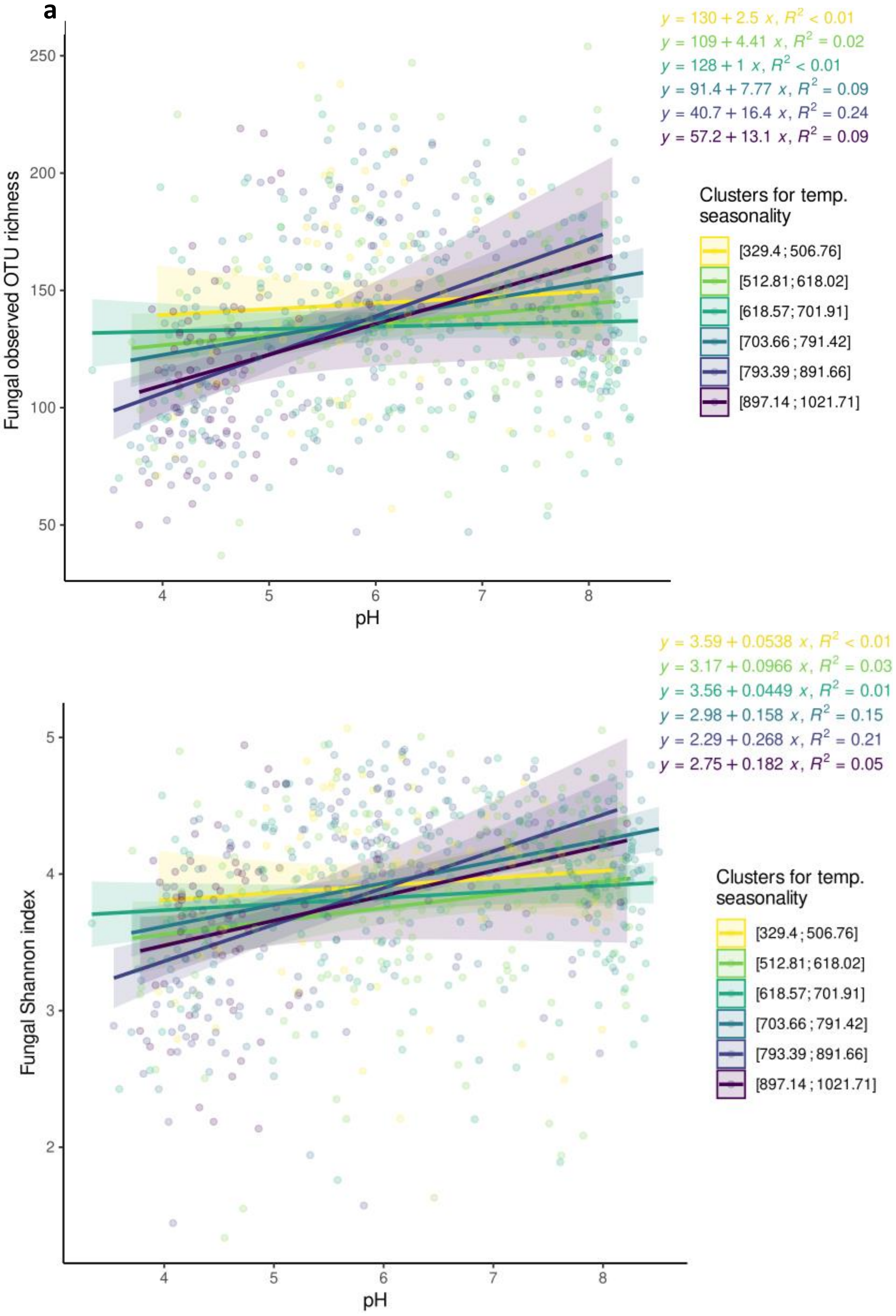
a
y = 130 + 2.5 x, R2 < 0.01
y = 109 + 4.41 x, R2 = 0.02
y = 128 + 1 x, R2 < 0.01
y = 91.4 + 7.77 x, R2 = 0.09
y = 40.7 + 16.4 x, R2 = 0.24
y = 57.2 + 13.1 x, R2 = 0.09
Fungal observed OTU richness
250
200
150
100
50
4
5
6
7
8
pH
Clusters for temp. seasonality
[329.4 ; 506.76]
[512.81 ; 618.02]
[618.57 ; 701.91]
[703.66 ; 791.42]
[793.39 ; 891.66]
[897.14 ; 1021.71]
y = 3.59 + 0.0538 x, R2 < 0.01
y = 3.17 + 0.0966 x, R2 = 0.03
y = 3.56 + 0.0449 x, R2 = 0.01
y = 2.98 + 0.158 x, R2 = 0.15
y = 2.29 + 0.268 x, R2 = 0.21
y = 2.75 + 0.182 x, R2 = 0.05
Fungal Shannon index
5
4
3
2
4
5
6
7
8
pH
Clusters for temp. seasonality
[329.4 ; 506.76]
[512.81 ; 618.02]
[618.57 ; 701.91]
[703.66 ; 791.42]
[793.39 ; 891.66]
[897.14 ; 1021.71]

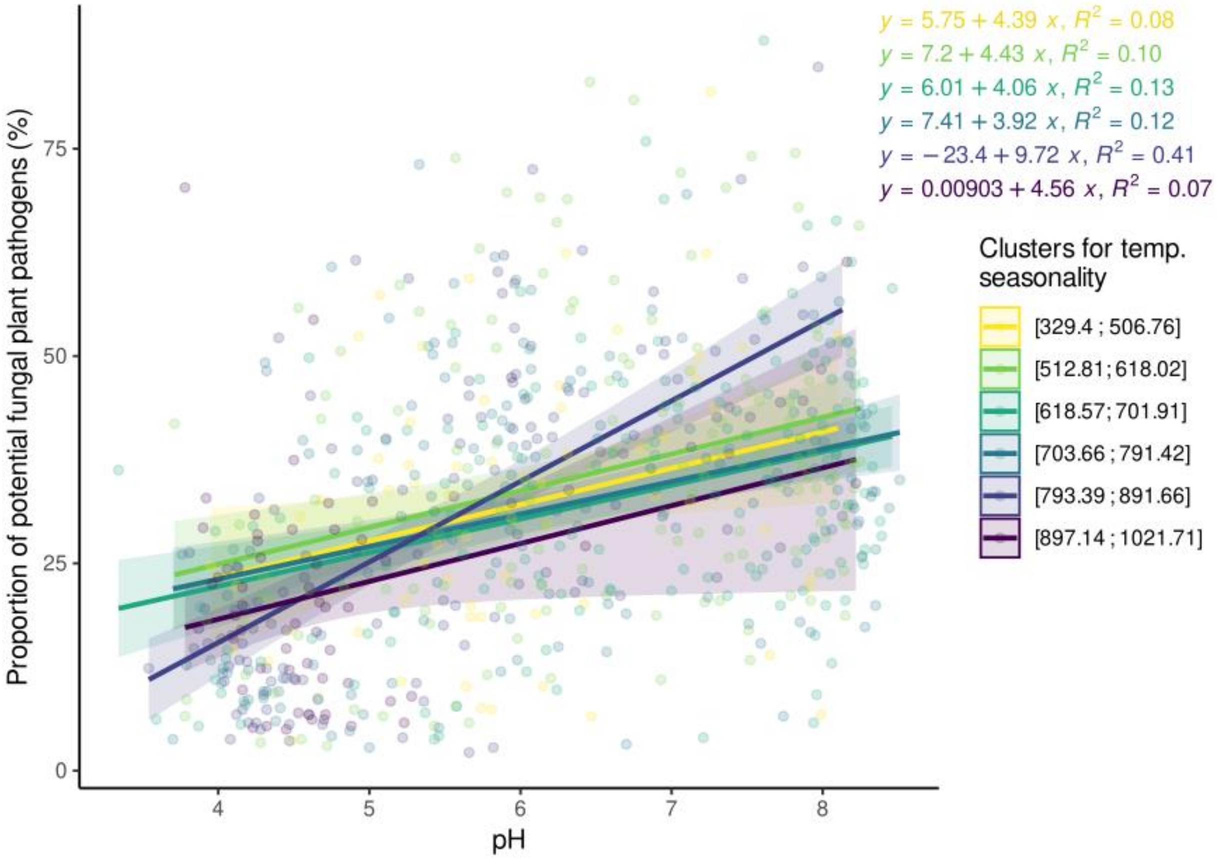

y = 5.75 + 4.39 x, R2 = 0.08
y = 7.2 + 4.43 x, R2 = 0.10
y = 6.01 + 4.06 x, R2 = 0.13
y = 7.41 + 3.92 x, R2 = 0.12
y = −23.4 + 9.72 x, R2 = 0.41
y = 0.00903 + 4.56 x, R2 = 0.07
Proportion of potential fungal plant pathogens (%)
0
25
50
75
4
5
6
7
8
pH
Clusters for temp. seasonality
[329.4 ; 506.76]
[512.81; 618.02]
[618.57; 701.91]
[703.66 ; 791.42]
[793.39 ; 891.66]
[897.14 ; 1021.71]

b

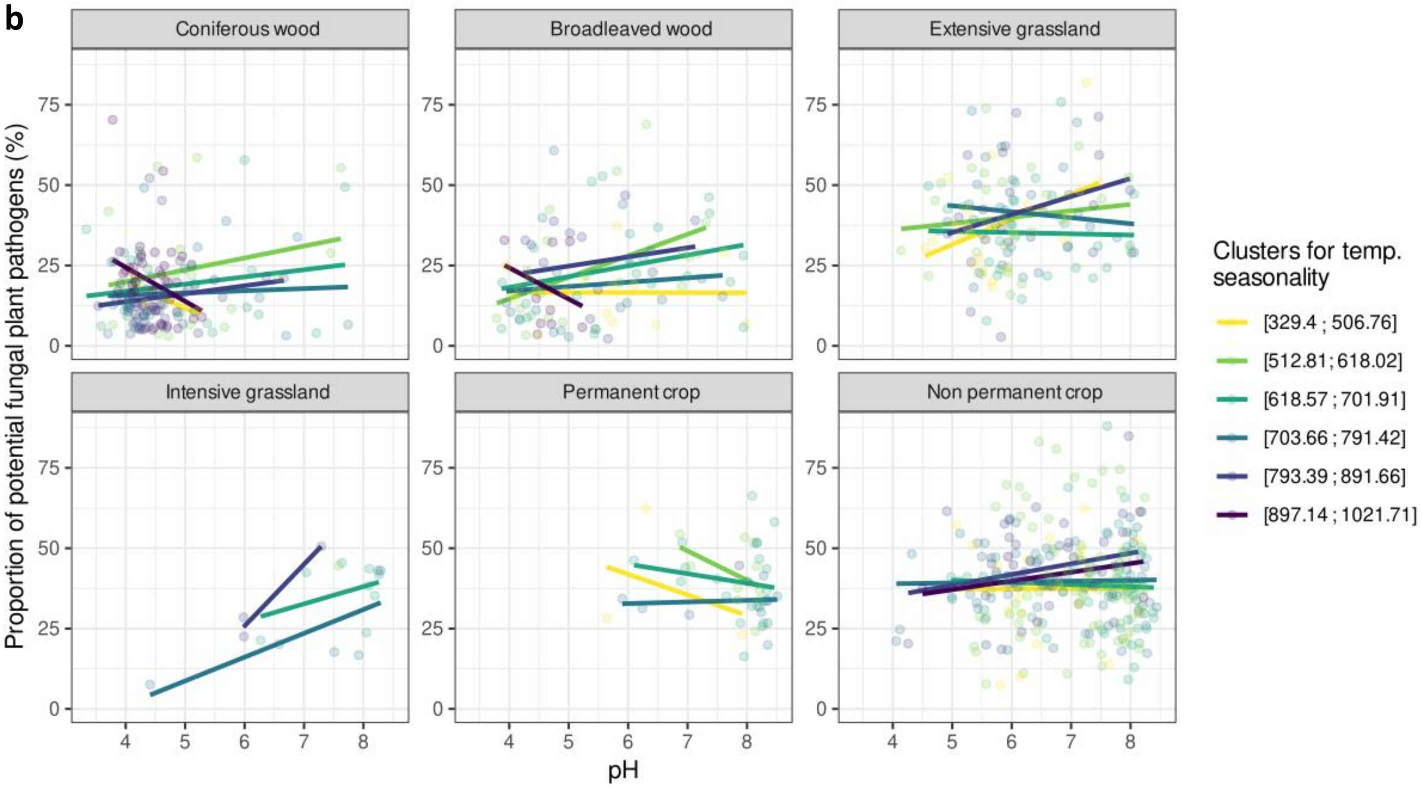

Supplementary Fig. 16.

Example of interaction plots. **a** Interaction plots among pH and temperature seasonality (standard deviation ×100) as highly influencing interactions for fungal observed OTU richness, fungal Shannon index and proportion of potential fungal pathogens (%), visualised all vegetation cover types together. Shaded areas represent the standard error. **b** Interaction plots among pH and temperature seasonality (standard deviation ×100) as highly influencing interactions for fungal observed OTU richness, fungal Shannon index and proportion of potential fungal pathogens (%), visualised separated by vegetation cover types for proportion of potential fungal pathogens. In all plots, clusters for temperature seasonality were created using k-means method for an optimal number of clusters (n=6). Each colour represents a given cluster. Source data are provided as a Source Data file.

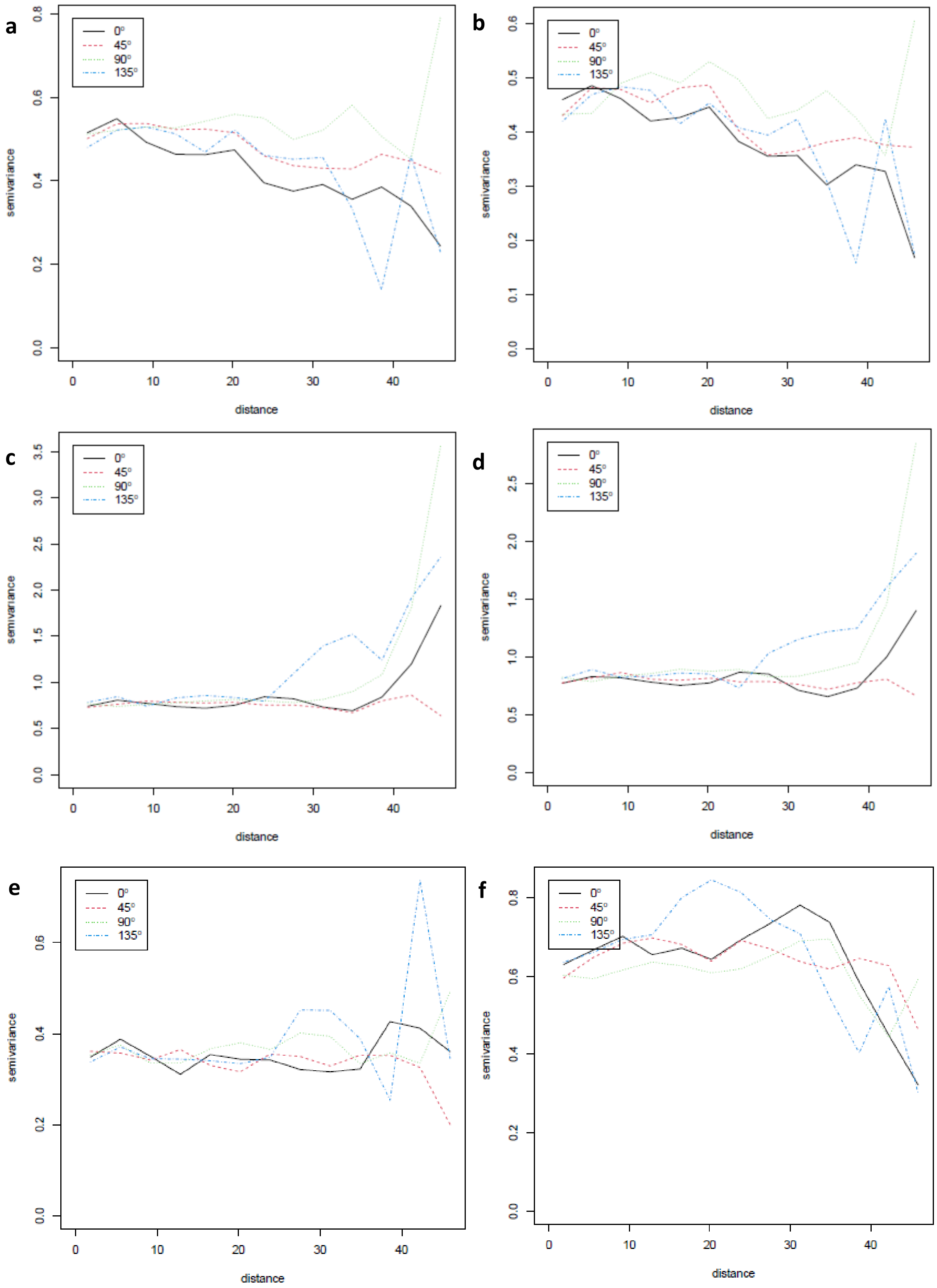
a
b
c
d
e
f
0°
45°
90°
135°
semivariance
distance

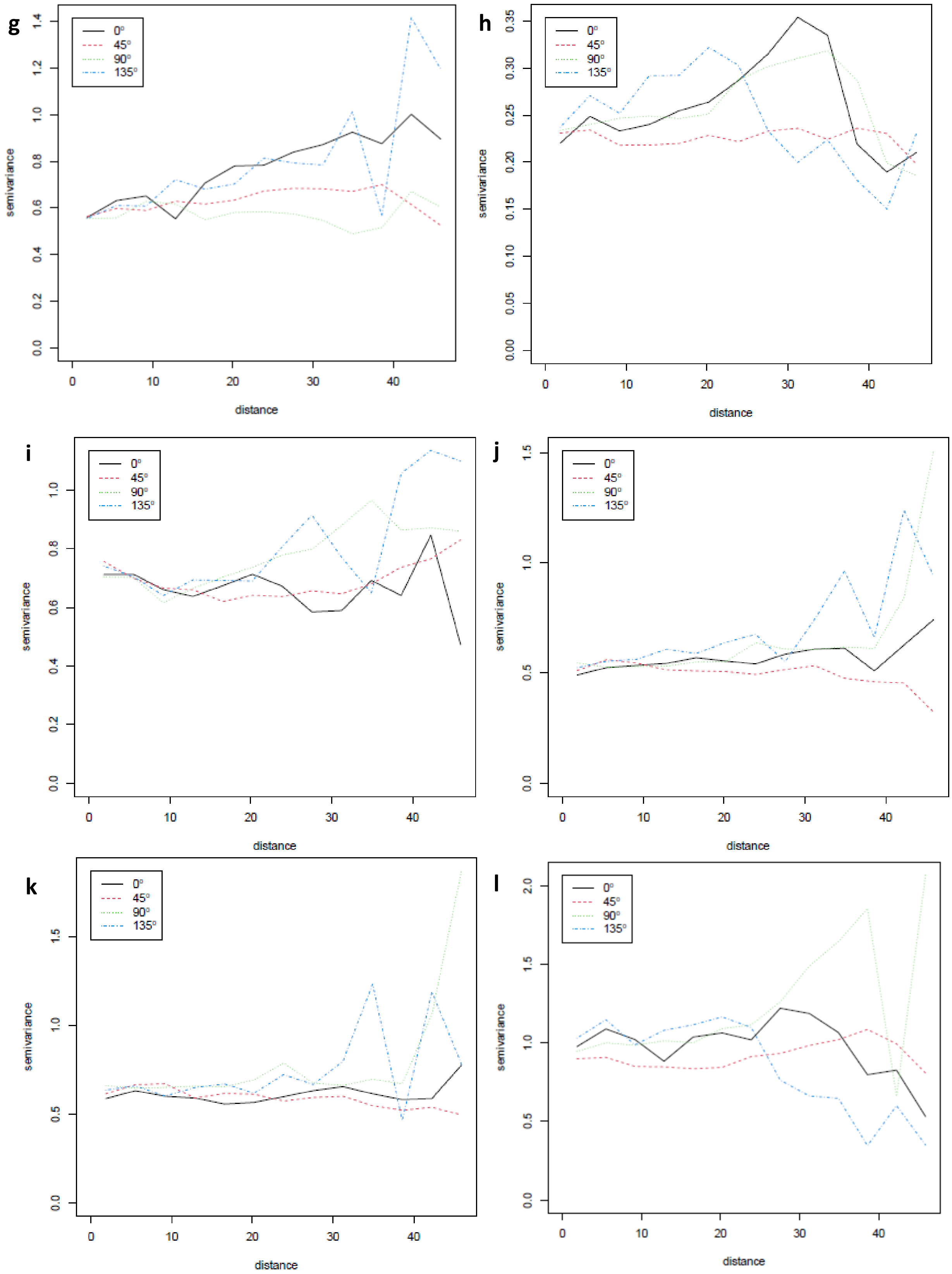
g
h
i
j
k
l
0°
45°
90°
135°
semivariance
distance

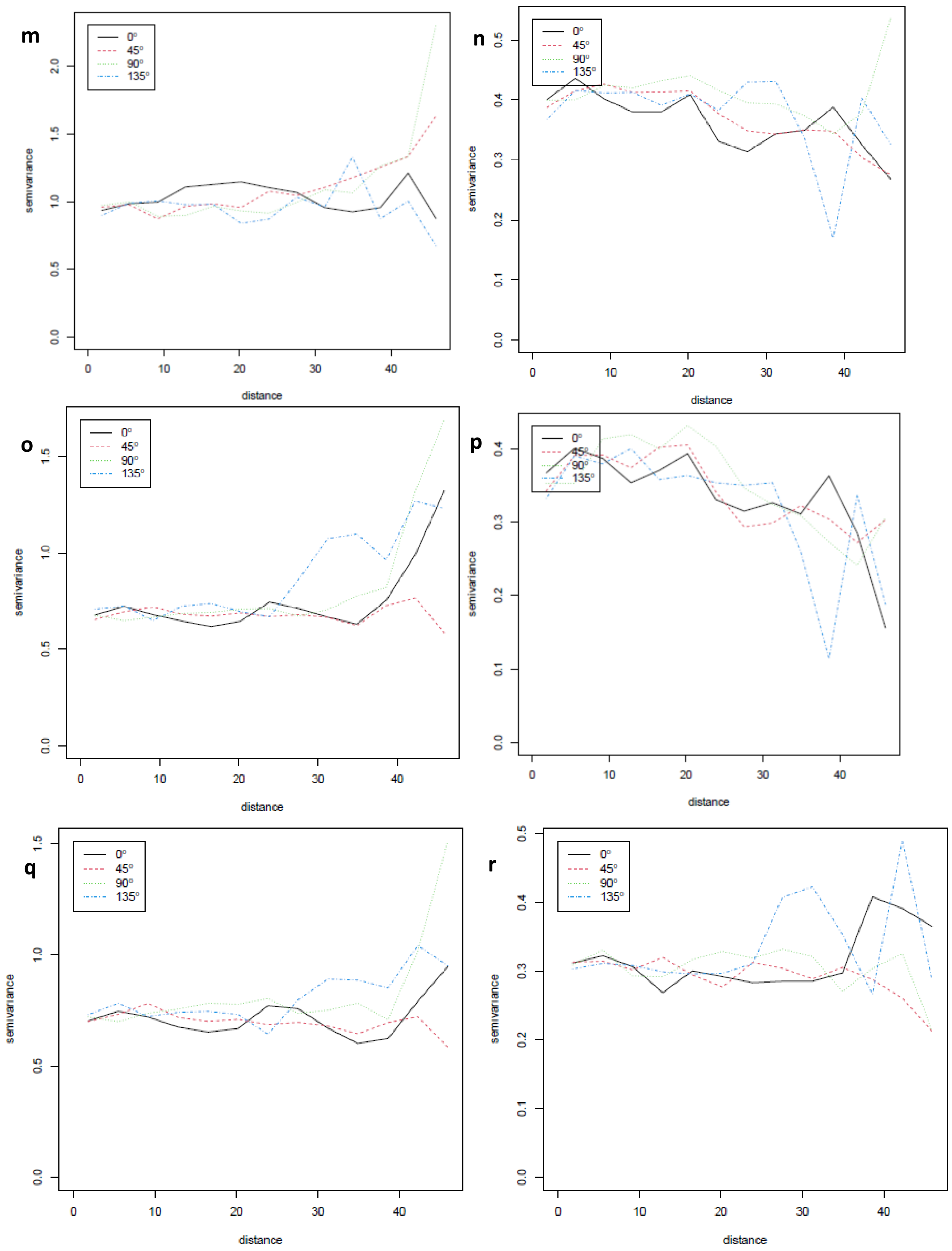
m
n
o
p
q
r
0°
45°
90°
135°
semivariance
distance

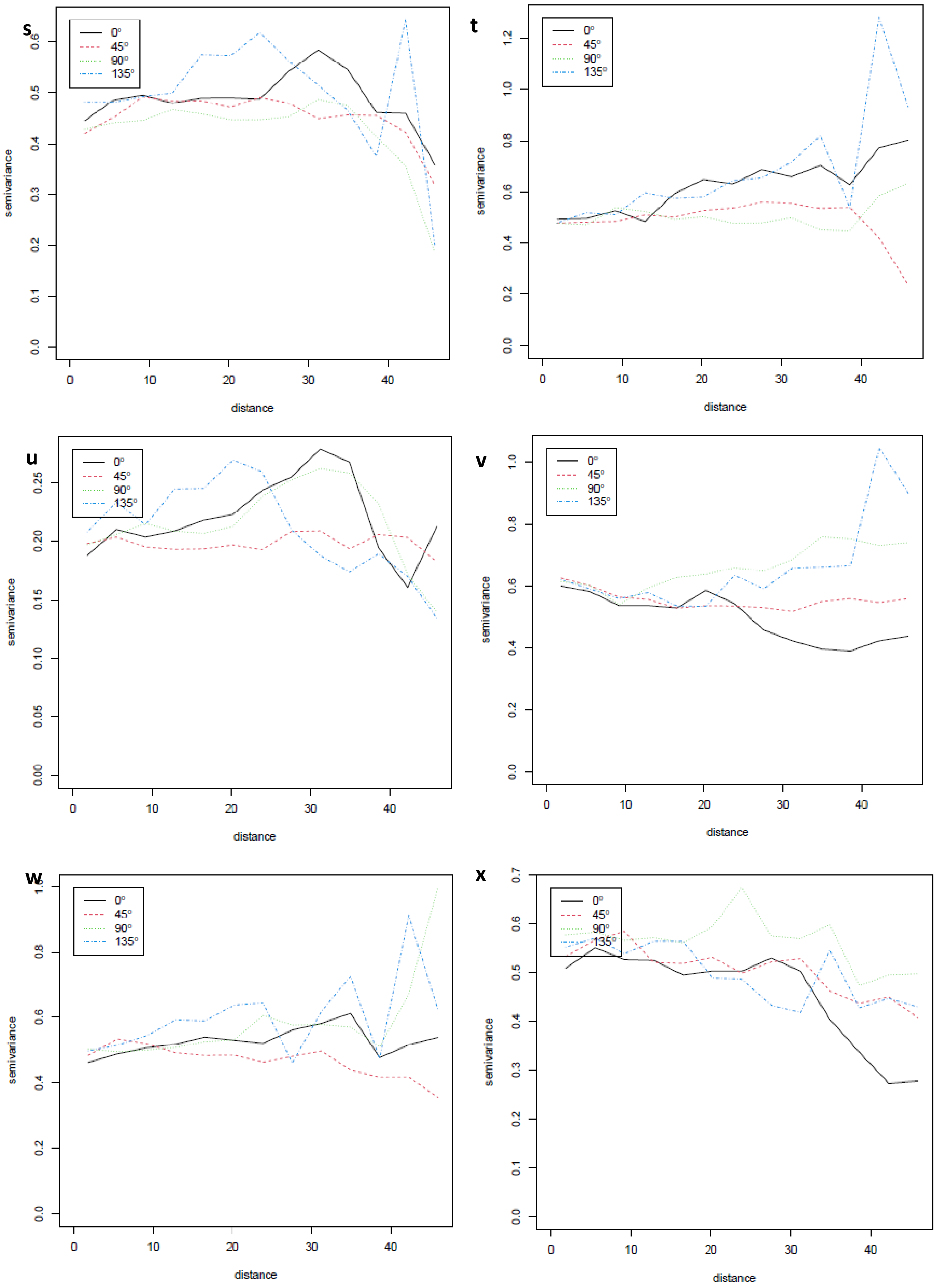
s
0°
45°
90°
135°
semivariance
distance
t
0°
45°
90°
135°
semivariance
distance
u
0°
45°
90°
135°
semivariance
distance
v
0°
45°
90°
135°
semivariance
distance
w
0°
45°
90°
135°
semivariance
distance
x
0°
45°
90°
135°
semivariance
distance

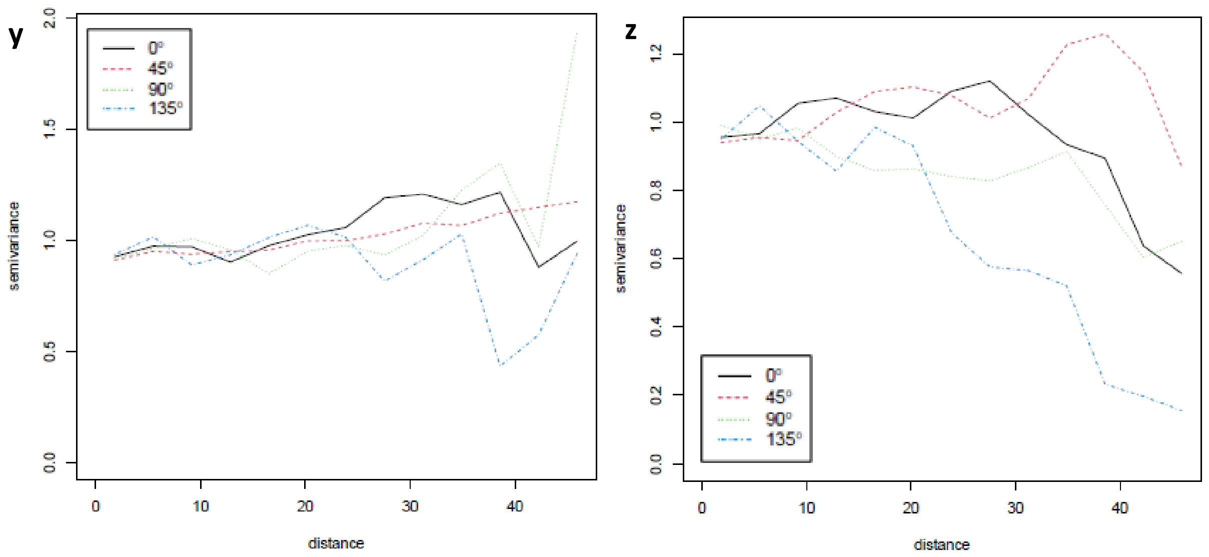

Supplementary Fig. 17.

Variograms for all models testing for soil properties, climate and vegetation as single effects on **a** bacterial observed richness, **b** fungal observed richness, **c** bacterial Shannon index, **d** fungal Shannon index, **e** bacterial chemoheterotrophs, **f** bacterial N-fixers, **g** bacterial pathogens, **h** ectomycorrhizal fungi, **i** arbuscular mycorrhizal fungi, **j** fungal saprotrophs, **k** fungal plant pathogens, **l** bacterial beta-diversity and **m** fungal beta-diversity, or as interaction effects on **n** bacterial observed richness, **o** fungal observed richness, **p** bacterial Shannon index, **q** fungal Shannon index, **r** bacterial chemoheterotrophs, **s** bacterial N-fixers, **t** bacterial pathogens, **u** ectomycorrhizal fungi, **v** arbuscular mycorrhizal fungi, **w** fungal saprotrophs and **x** fungal plant pathogens, **y** bacterial beta-diversity and **z** fungal beta-diversity. Distance on the x-axis is expressed in degrees.

Supplementary Table 1.

Values taken by the bacterial and fungal observed richness and Shannon index depending on the vegetation cover type (mean ± SD).

	Observed richness		Shannon index	
Vegetation cover	Bacteria	Fungi	Bacteria	Fungi
1.Coniferous wood	5840 ± 1613	110 ± 31	7.19 ± 0.52	3.41 ± 0.64
2.Broadleaved wood	7321 ± 1991	130 ± 39	7.59 ± 0.58	3.77 ± 0.60
3.Extensive grassland	8091 ± 1344	156 ± 39	7.82 ± 0.44	4.06 ± 0.67
4.Intensive grassland	8301 ± 958	137 ± 26	7.96 ± 0.28	3.96 ± 0.48
5.Permanent crop	8647 ± 1176	140 ± 36	8.00 ± 0.28	3.99 ± 0.44
6.Non permanent crop	8764 ± 1428	143 ± 33	8.02 ± 0.36	3.99 ± 0.58

Supplementary Table 2.

Mean relative abundance of the ten most abundant bacterial and fungal phyla and classes at the community-level.

Bacteria

Phylum	Mean relative abundance	Class	Mean relative abundance
Proteobacteria	0.306	Actinobacteria	0.254
Actinobacteria	0.261	Alphaproteobacteria	0.174
Acidobacteria	0.229	Acidobacteria Grp 1	0.0621
Verrucomicrobia	0.0547	Acidobacteria Grp 6	0.0541
Bacteroidetes	0.0492	Gammaproteobacteria	0.0454
Planctomycetes	0.0412	Betaproteobacteria	0.0446
Chloroflexi	0.0159	Acidobacteria Grp 2	0.0411
candidate_division_WPS-1	0.0134	Planctomycetia	0.0411
Firmicutes	0.0116	Deltaproteobacteria	0.0349
Gemmatimonadetes	0.00418	Sphingobacteriia	0.0298

Fungi

Phylum	Mean relative abundance	Class	Mean relative abundance
Ascomycota	0.493	Agaricomycetes	0.287
Basidiomycota	0.341	Sordariomycetes	0.167
Mortierellomycota	0.0786	Leotiomycetes	0.105
Chytridiomycota	0.0269	Dothideomycetes	0.102
Mucoromycota	0.0222	Mortierellomycetes	0.0784
Rozellomycota	0.0151	Pezozimycetes	0.0459
Glomeromycota	0.00805	Eurotiomycetes	0.0456
Unknown	0.00483	Tremellomycetes	0.0381
Zoopagomycota	0.00433	Mucoromycetes	0.0135
Blastocladiomycota	0.00151	Spizellomycetes	0.00659

Supplementary Table 3.
Mean relative abundance of bacterial phyla and classes at the functional group-level. Only the ten most abundant phyla and classes are displayed for bacterial chemoheterotrophs.

	Phylum	Mean relative abundance	Class	Mean relative abundance
Bacterial chemoheterotrophs	Actinobacteria	0.521	Actinobacteria	0.521
	Proteobacteria	0.345	Alphaproteobacteria	0.232
	Bacteroidetes	0.0375	Betaproteobacteria	0.0762
	Verrucomicrobia	0.0318	Opitutae	0.0297
	Gemmatimonadetes	0.0177	Gammaproteobacteria	0.0283
	Planctomycetes	0.0191	Planctomycetia	0.0191
	Acidobacteria	0.0144	Flavobacteriia	0.0186
	Firmicutes	0.0091	Gemmatimonadetes	0.0177
	Chloroflexi	0.0038	Cytophagia	0.011
	Spirochaetes	0.00054	Acidobacteria grp1	0.0105
	Ignavibacteriae	0.000372		
Bacterial N-fixers	Proteobacteria	0.988	Alphaproteobacteria	0.977
	Firmicutes	0.0117	Clostridia	0.0117
			Deltaproteobacteria	0.00718
			Betaproteobacteria	0.00307
			Gammaproteobacteria	0.000858
Bacterial pathogens	Actinobacteria	0.459	Actinobacteria	0.459
	Proteobacteria	0.352	Alphaproteobacteria	0.189
	Firmicutes	0.189	Bacilli	0.181
			Gammaproteobacteria	0.105
			Betaproteobacteria	0.0664

Supplementary Table 4.

Mean relative abundance of fungal phyla and classes at the functional group-level. Only the ten most abundant phyla and classes are displayed for fungal saprotrophs and plant pathogens.

	Phylum	Mean relative abundance	Class	Mean relative abundance
EcM	Basidiomycota	0.891	Agaricomycetes	0.891
	Ascomycota	0.106	Pezizomycetes	0.0902
	Mucoromycota	0.00275	Dothideomycetes	0.0107
			Eurotiomycetes	0.0049
			Endogonomycetes	0.00275
AMF	Glomeromycota	1	Glomeromycetes	0.705
			Paraglomeromycetes	0.18
			Archaeosporomycetes	0.12
			Unknown class	0.00214
Fungal saprotrophs	Ascomycota	0.568	Sordariomycetes	0.173
	Basidiomycota	0.245	Agaricomycetes	0.17
	Mortierellomycota	0.13	Leotiomycetes	0.152
	Mucoromycota	0.0395	Mortierellomycetes	0.13
	Chytridiomycota	0.0161	Dothideomycetes	0.122
	Basidiobolomycota	0.000485	Tremellomycetes	0.0624
	Kickxellomycota	0.000473	Eurotiomycetes	0.0601
	Calcarisporiellomycota	0.000435	Pezizomycetes	0.0424
	Monoblepharomycota	0.000184	Mucoromycetes	0.019
	Blastocladiomycota	0.0000573	Umbelopsidomycetes	0.019
Fungal plant pathogens	Ascomycota	0.564	Mortierellomycetes	0.234
	Mortierellomycota	0.234	Sordariomycetes	0.203
	Basidiomycota	0.143	Dothideomycetes	0.169
	Mucoromycota	0.0374	Agaricomycetes	0.134
	Chytridiomycota	0.0164	Leotiomycetes	0.129
	Olpidiomycota	0.00233	Eurotiomycetes	0.0435
	Enthorrhizomycota	0.00217	Umbelopsidomycetes	0.0333
	Monoblepharomycota	0.000806	Archaeorhizomycetes	0.0159
			Rhizophlyctidomycetes	0.00905
			Mucoromycetes	0.0047

Supplementary Table 5.

Model performance for single- and interaction-effect models. Adjusted R^2 (%) and Akaike Information Criteria (AIC) values of the ordinary least squares models (or dbRDA ordination, for β-diversity) testing the single effect of soil properties, climate and vegetation cover or the interaction effect between soil properties, climate and vegetation cover for the bacterial and fungal α- and β-diversity and functional groups. Lower AIC values indicate more parsimonious models.

		Single-effect model (or ordination)		Interaction model (or ordination)	
		Adjusted R^2	AIC	Adjusted R^2	AIC
Observed richness	Bacteria	48.89	-463.41	56.43	-531.57
	Fungi	20.35	-149.79	26.04	-167.2
Shannon index	Bacteria	54.37	-543.47	60.46	-612.79
	Fungi	16.79	-119.56	20.96	-1188
B-diversity	Bacteria	37.34	3573.29	40.15	3626.96
	Fungi	12.79	4051.07	15.59	4106.00
Chemoheterotrophs	Bacteria	64.19	-719.79	67.65	-766.6
N-fixers	Bacteria	32.61	-266.41	48.41	-419.57
Human pathogens	Bacteria	35.36	-298.15	43.74	-355.89
Ectomycorrhiza	Fungi	65.91	-976.97	68.67	-993.68
Arbuscular mycorrhiza	Fungi	29.57	-236.83	36.75	-263.03
Saprotrophs	Fungi	44.47	-405.22	46.56	-421.07
Plant pathogens	Fungi	35.81	-301.19	42.19	-345.5

Supplementary Table 6.

ISO standards and references for soil properties.

Soil property	ISO standard	Reference
Bulk density	x	x
Clay and silt contents	13320:2009	ISO (International Organization for Standardization) 2009. ISO 13320:2009 Particle Size Analysis Laser Diffraction Methods. International Organization for Standardization, Geneva
Coarse fragment	x	x
Calcium carbonate content	10693:1995	ISO (International Organization for Standardization) 1995. ISO 10693:1995 Soil Quality Determination of Carbonate Content Volumetric Method. International Organization for Standardization, Geneva
Extractable potassium content	x	x
pH (H2O)	10390:1994	ISO (International Organization for Standardization) 1994. ISO 10390:1994 Soil Quality Determination of pH. International Organization for Standardization, Geneva.
Available phosphorus content	11263:1994	ISO (International Organization for Standardization) 1994. ISO 11263:1994 Soil Quality Determination of Phosphorus Spectrometric Determination of Phosphorus Soluble in Sodium Hydrogen Carbonate Solution. International Organization for Standardization, Geneva
Organic carbon	10694:1995	ISO (International Organization for Standardization) 1995. ISO 10694:1995 Soil Quality Determination of Organic and Total Carbon after Dry Combustion (Elementary Analysis). International Organization for Standardization, Geneva
Total nitrogen content	11261:1995	ISO (International Organization for Standardization) 1995. ISO 11261:1995 Soil Quality Determination of Total Nitrogen Modified Kjeldahl Method. International Organization for Standardization, Geneva.

Supplementary Table 7.

Transformation of explained variable.

	Transformation	Parameters
Observed richness 16S	orderNorm*	
Observed richness ITS	Standardized sqrt(x + a)	a = 0
Shannon index 16S	orderNorm	
Shannon index ITS	Standardized Yeo-Johnson	lambda = 3.051889
Bacterial chemoheterotrophs	orderNorm	
Bacterial N-fixers	Standardized Box Cox	lambda = 0.3459031
Bacterial pathogens	orderNorm	
EMF	orderNorm	
AMF	Standardized sqrt(x + a)	a = 0
Fungal saprotrophs	orderNorm	
Fungal plant pathogens	Standardized asinh(x)	

*for more details on the orderNorm transformation, see: https://www.rdocumentation.org/packages/bestNormalize/versions/1.8.2/topics/orderNorm
