# Supplementary material for: Patterns in soil microbial diversity across Europe
Source: Nat Commun. 2023 Jun 8;14:3311. doi: 10.1038/s41467-023-37937-4 (PMC10250377; doi:10.1038/s41467-023-37937-4)
Supplement: Supplementary file 2 — Reporting Summary [file 41467_2023_37937_MOESM2_ESM.pdf]

nature portfolio

Corresponding author(s): Alberto Orgiazzi, Marcel van der Heijden

Last updated by author(s): Mar 27, 2023

Reporting Summary

Nature Portfolio wishes to improve the reproducibility of the work that we publish. This form provides structure for consistency and transparency in reporting. For further information on Nature Portfolio policies, see our Editorial Policies and the Editorial Policy Checklist.

Statistics

For all statistical analyses, confirm that the following items are present in the figure legend, table legend, main text, or Methods section.

n/a	Confirmed	
☐	☒	The exact sample size (n) for each experimental group/condition, given as a discrete number and unit of measurement
☐	☒	A statement on whether measurements were taken from distinct samples or whether the same sample was measured repeatedly
☐	☒	The statistical test(s) used AND whether they are one- or two-sided<br>*Only common tests should be described solely by name; describe more complex techniques in the Methods section.*
☐	☒	A description of all covariates tested
☐	☒	A description of any assumptions or corrections, such as tests of normality and adjustment for multiple comparisons
☐	☒	A full description of the statistical parameters including central tendency (e.g. means) or other basic estimates (e.g. regression coefficient) AND variation (e.g. standard deviation) or associated estimates of uncertainty (e.g. confidence intervals)
☐	☒	For null hypothesis testing, the test statistic (e.g. F, t, r) with confidence intervals, effect sizes, degrees of freedom and P value noted<br>*Give P values as exact values whenever suitable.*
☒	☐	For Bayesian analysis, information on the choice of priors and Markov chain Monte Carlo settings
☒	☐	For hierarchical and complex designs, identification of the appropriate level for tests and full reporting of outcomes
☒	☐	Estimates of effect sizes (e.g. Cohen's d, Pearson's r), indicating how they were calculated

Our web collection on statistics for biologists contains articles on many of the points above.

Software and code

Policy information about availability of computer code

Data collection

We used Rstudio (version 2021.09.0) and R (version 4.2.1).

Data analysis

We used Rstudio (version 2021.09.0) and R (version 4.2.1). All R scripts used for the analyses are available on the European Soil Data Centre (https://esdac.jrc.ec.europa.eu/content/soil-biodiversity-dna-bacteria-and-fungi).

The R packages needed are the following:
#library(devtools) v2.4.3.
#library(BiocManager) v1.30.18
library(tidyr) v1.2.0
library(treeio) v1.20.0 – installation requires the package BiocManager (see here : https://bioconductor.org/packages/release/bioc/html/treeio.html)
library(phyloseq) v1.34.0 – installation requires the package BiocManager (see here : https://www.bioconductor.org/packages/release/bioc/html/phyloseq.html)
library(ggplot2) v3.3.5
library(stringr) v1.4.0
library(multcompView) v0.1.8
library(dplyr) v1.0.8
library(vegan) v2.5.7
library(sf) v1.0.7
library(SRS) v0.2.3

library(readxl) v1.4.0
library(plyr) v1.8.7
library(microeco) v0.11.0
library(magrittr) v2.0.3
library(imputeTS) v3.2
library(missMDA) v1.18
library(lmtest) v0.9.39
library(sandwich) v3.0.1
library(geoR) v1.9.2
library(pairwiseAdonis) v0.4 – installation requires the package devtools and function install_github() (see here : https://github.com/pmartinezarbizu/pairwiseAdonis)
library(car) v3.0.12
library(ggord) v1.1.5 – instructions of installation here: https://fawda123.github.io/ggord/
library(ggordiplots) v0.4.1 – instructions of installation here: https://rdrr.io/github/jfq3/ggordiplots/

For manuscripts utilizing custom algorithms or software that are central to the research but not yet described in published literature, software must be made available to editors and reviewers. We strongly encourage code deposition in a community repository (e.g. GitHub). See the Nature Portfolio guidelines for submitting code & software for further information.

Data

Policy information about availability of data

All manuscripts must include a data availability statement. This statement should provide the following information, where applicable:

- Accession codes, unique identifiers, or web links for publicly available datasets
- A description of any restrictions on data availability
- For clinical datasets or third party data, please ensure that the statement adheres to our policy

The raw data and sampling site metadata used in this study are available on the European Soil Data Centre (https://esdac.jrc.ec.europa.eu/content/soil-biodiversity-dna-bacteria-and-fungi). The data generated in this study are provided in the Supplementary Information, Supplementary Data 1 and 2, and Source Data file. Source data are provided with this paper.

This study also used online databases/datasets:
- WordClim - Historical monthly weather data. https://worldclim.org/data/monthlywth.html
- Global Aridity Index and Potential Evapotranspiration Climate Database v2. https://doi.org/10.6084/m9.figshare.7504448.v3
- NASA-USDA Enhanced SMAP Global Soil Moisture Data. https://developers.google.com/earth-engine/datasets/catalog/NASA_USDA_HSL_SMAP10KM_soil_moisture#description
- Biogeographical regions from the European Environmental Agency (EEA), downloadable at https://www.eea.europa.eu/data-and-maps/data/biogeographical-regions-europe-3 (shapefile)
- FungalTraits (Põlme, S. et al. FungalTraits: a user-friendly traits database of fungi and fungus-like stramenopiles. Fungal Diversity 105, 1–16 (2020)).
- FAPROTAX (Louca, S., Parfrey, L. W. & Doebeli, M. Decoupling function and taxonomy in the global ocean microbiome. Science 353, 1272–1277 (2016)).
FungalTraits can be downloaded from the UNITE homepage https://unite.ut.ee/repository.php. FAPROTAX database is freely available at http://www.loucalab.com/archive/FAPROTAX/.

Human research participants

Policy information about studies involving human research participants and Sex and Gender in Research.

Reporting on sex and gender	*this does not apply to this study*
Population characteristics	*this does not apply to this study*
Recruitment	*this does not apply to this study*
Ethics oversight	*this does not apply to this study*

Note that full information on the approval of the study protocol must also be provided in the manuscript.

Field-specific reporting

Please select the one below that is the best fit for your research. If you are not sure, read the appropriate sections before making your selection.

☐ Life sciences ☐ Behavioural & social sciences ☒ Ecological, evolutionary & environmental sciences

For a reference copy of the document with all sections, see nature.com/documents/nr-reporting-summary-flat.pdf

Ecological, evolutionary & environmental sciences study design

All studies must disclose on these points even when the disclosure is negative.

Study description	We compared microbial communities in six vegetation cover types and aimed to determine the main drivers (soil properties, climate, vegetation) and their single- and interaction-effects on soil microbial assemblages, but also, for the first time, on potential functions associated to the microbial communities.
Research sample	We analysed bacterial and fungal DNA sequences from 715 soil samples collected all over Europe as part of LUCAS (Land Use/Cover Area frame Survey), the largest European soil survey coordinated by the European Commission.
Sampling strategy	Sampling points were selected based on a simulated annealing sampling applied to the LUCAS Soil 2009 data as the original population from which to sample. Point selection was performed so that an optimal configuration was found replicating a wide range of environmental variables (i.e., soil physico-chemical properties, topography, climate and land cover).
Data collection	At each location, five subsamples covering a depth of 20 cm were collected and mixed together. One subsample was collected at the precise geographical location of the pre-selected point while four additional subsamples were collected at the four cardinal directions (North, East, South and West), at a distance of 2m from the first subsample location in each direction. Data collection was performed by trained surveyors. Surveyor instructions for data collection are presented in Fernández-Ugalde O., Orgiazzi A., Jones A., Lugato E., Panagos P., LUCAS 2018 – SOIL COMPONENT: Sampling Instructions for Surveyors, EUR 28501 EN, doi 10.2760/023673 https://esdac.jrc.ec.europa.eu/public_path/shared_folder/doc_pub/JRC105923_LUCAS2018_JRCTechnicalReport.pdf
Timing and spatial scale	Data collection took place across Europe from April to December 2018. The final 715 selected soil samples were part of 24 European countries and 699 of them (>97%) were sampled between May and October.
Data exclusions	For statistical reasons, we focused our study on sites belonging to three main land covers (cropland, grassland, woodland) in four biogeographical (climatic) regions, allowing to compare the effect of vegetation types of interest among the same wide biogeographical conditions. Only the sites presenting enough fungal data (in terms of read counts) for the normalization step were kept, and the same sites were selected for bacterial data to allow comparisons between microorganism types in the exact same environmental conditions. Archaeal, chloroplasts and mitochondrial zOTUs were removed from the 16S dataset, which collectively accounted for 0.34% of all zOTUs.
Reproducibility	To date, there is no repeat of the experiment, but a LUCAS biodiversity campaign is ongoing this year 2022 and will allow in the future to verify the patterns found for 2018. Analysis and results for the 2022 campaign will be available in 2025.
Randomization	Samples were characterized by their meta-information (land cover, country, biogeographical (climatic) region) but not allocated into further groups for the analyses.
Blinding	*Describe the extent of blinding used during data acquisition and analysis. If blinding was not possible, describe why OR explain why blinding was not relevant to your study.*

Did the study involve field work? ☒ Yes ☐ No

Field work, collection and transport

Field conditions	Soil samples were collected from April to December 2018. A full list of metadata (e.g., date, coordinates) were collected. Climatic conditions were accounted for into the models.
Location	Soils samples were collected across European Union and United Kingdom. Soils samples were collected across European Union and United Kingdom. Sampling locations extended from Sweden to Cyprus (latitudinal gradient) and from Portugal to Cyprus (longitudinal gradient). Maximal elevation was 1800m.
Access & import/export	Samples were collected following national rules. In some cases (private land) owners were duly informed upon collection. No permission was sought for land in public ownership.
Disturbance	No disturbance were implied.

Reporting for specific materials, systems and methods

We require information from authors about some types of materials, experimental systems and methods used in many studies. Here, indicate whether each material, system or method listed is relevant to your study. If you are not sure if a list item applies to your research, read the appropriate section before selecting a response.

Materials & experimental systems

n/a	Involved in the study
☒	☐ Antibodies
☒	☐ Eukaryotic cell lines
☒	☐ Palaeontology and archaeology
☒	☐ Animals and other organisms
☒	☐ Clinical data
☒	☐ Dual use research of concern

Methods

n/a	Involved in the study
☒	☐ ChIP-seq
☒	☐ Flow cytometry
☒	☐ MRI-based neuroimaging
