# Supplementary material for: Patterns in soil microbial diversity across Europe
Source: Nat Commun. 2023 Jun 8;14:3311. doi: 10.1038/s41467-023-37937-4 (PMC10250377; doi:10.1038/s41467-023-37937-4)
Supplement: Supplementary file 4 — Description of Additional Information file [file 41467_2023_37937_MOESM4_ESM.pdf]

File Name: Supplementary Data 1

Description: This file includes values taken by the observed fungal OTU richness and Shannon index among different normalization thresholds across common (i.e., non-discarded) sites. These values illustrate the high correlation (r^2>0.92) between richness/Shannon index at different thresholds and justify the use of a normalization threshold at 502 read counts for fungi, that permits to compare both bacterial and fungal data on a larger number of sampling sites.

File Name: Supplementary Data 2

Description: This file includes one-way ANOVA and permuted ANOVA (PERMANOVA) outputs for the alpha- and beta-diversity models. For alpha-diversity analyses, p-values obtained with a PERMANOVA were compared to the ones from a regular one-way ANOVA, were found consistent between methods, and were added as significance stars to the figures representing the variable importance of each variable in the models (Figures 4 and 5 of the manuscript and Supplementary figures 7 and 12). For beta-diversity analyses, p-values from PERMANOVA performed on the ordinations were added as significance stars to the figures representing the variable importance of each variable in the models (Supplementary figures 8 and 12).
